# Lyophilized and Oven-Dried *Manilkara zapota* Extracts: Characterization and *In Vitro*, *In Vivo*, and *In Silico* Analyses

**DOI:** 10.3390/plants14020216

**Published:** 2025-01-14

**Authors:** María Fernanda Rivas-Gastélum, Patricia Ariadna Galindo-Castillo, Juan Esparza-Sánchez, Miriam Irene Jiménez-Pérez, Yocanxóchitl Perfecto-Avalos, Luis Eduardo Garcia-Amezquita, Diego E. Navarro-López, Edgar R. López-Mena, Eugenio Sánchez-Arreola, Juan Pablo Tamayo-Martínez, Humberto L. Mendoza-Figueroa, María Magdalena Crosby-Galván, Elsa Margarita Crosby-Galván, Jorge L. Mejía-Méndez, Angélica Lizeth Sánchez-López

**Affiliations:** 1 Tecnologico de Monterrey, Escuela de Ingeniería y Ciencias, Av. Gral. Ramón Corona No 2514, Colonia Nuevo México, Zapopan 45121, Mexico; rivas0898@gmail.com (M.F.R.-G.); a01641610@tec.mx (P.A.G.-C.); a01638675@tec.mx (J.E.-S.); miriamjim@tec.mx (M.I.J.-P.); yocan@tec.mx (Y.P.-A.); garcia.amezquita@tec.mx (L.E.G.-A.); diegonl@tec.mx (D.E.N.-L.); edgarl@tec.mx (E.R.L.-M.); 2 Departamento de Ciencias Químico Biológicas, Universidad de las Américas Puebla, Santa Catarina Mártir s/n, San Andrés Cholula 72810, Mexico; eugenio.sanchez@udlap.mx; 3 Laboratorio de Diseño y Desarrollo de Nuevos Fármacos e Innovación Biotecnológica, Escuela Superior de Medicina, Instituto Politécnico Nacional, Plan de San Luis y Díaz Mirón s/n, Casco de Santo Tomás, Ciudad de México 11340, Mexico; jtamayom2300@alumno.ipn.mx (J.P.T.-M.).; hmendozaf@ipn.mx (H.L.M.-F.); 4 Programa de Ganadería, Colegio de Postgraduados, Campus Montecillo, Carretera México-Texcoco km 36.5, Texcoco 56264, Mexico; maria@colpos.mx (M.M.C.-G.); crosby.elsa@colpos.mx (E.M.C.-G.); 5 Programa de Edafología, Colegio de Postgraduados, Campus Montecillo, Carr. México Texcoco km 36.4, Montecillo 56264, Mexico

**Keywords:** chicozapote, drying methods, biological properties, antibacterial activity, antioxidant capacity

## Abstract

In this work, extracts from the pulp, peel, and seed of *Manilkara zapota* were obtained via lyophilization and oven drying. Bromatological analyses were performed to investigate variabilities in the nutritional content of fruits after nine post-harvest days. The phytochemical content of fruits was assessed by gas chromatography flame ionization detector (GC-FID), and their biological performance was studied using *in vitro* antibacterial and antioxidant assays (DPPH and ABTS) and *in vivo* toxicity models. Molecular docking was implemented to evaluate the interaction between polar compounds from chicozapote fruits with receptors involved in the pathogenesis of bacterial strains. Results revealed that water or soluble solids content did not vary after post-harvest. It was demonstrated that lyophilization or oven-drying approaches influenced the insoluble, total dietary fiber and digestible carbohydrates among samples. According to GC-FID analysis, it was observed that lyophilization and oven-drying methods also altered the content of myristic and pentadecanoic acids among the obtained extracts. It was noted that the antibacterial and antioxidant activities of extracts were weak due to their MIC (>1000 μg/mL) and IC_50_ (>2000 μg/mL) values. Still, the toxicity of extracts was poor against *Artemia salina* nauplii. *In silico* evaluation unveiled that polar compounds in *M. zapota* fruits possess a high binding affinity towards the DNA gyrase B of the cultured strains. This study expands the scientific evidence regarding the influence of distinct extraction methods on the nutritional and nutraceutical content of native fruits and the importance of considering additional approaches to enhance their bioactivities.

## 1. Introduction

*Manilkara zapota*, frequently known as chicozapote, is an evergreen fruit tree belonging to the family *Sapotaceae* [1]. Originally, the term chicozapote was designated from Nahuatl, a major Indigenous dialect in Mexico, but is also referred to as sapodilla, naseberry, sopota, or chikoo [2]. Geographically, chicozapote fruits are native to areas adjacent to southern Mexico and Central America. In Mexico, chicozapote fruits are predominantly cultivated in tropical and subtropical climates with warm temperatures, high humidity, and soils with adequate acidity and content of organic matter. Some of these regions include Oaxaca [3], Tabasco [4], and Yucatan [5].

Contrarily to other fruits, chicozapote fruits are characterized by their round or oval appearance, ranging from 5 to 9 cm in diameter, and weighing between 70 and 300 g [6]. The peel of chicozapote fruits is brown and rough when mature, whereas their pulp is fleshy and juicy, with a reddish-brown color. The seeds are black and shiny, with an average of 1–5 seeds per fruit [7]. Economically, chicozapote fruits are poorly commercialized since they exhibit a maximum shelf life of 11 days [8], converting them into highly perishable products despite their high nutritional and phytochemical content, and broad traditional medicinal uses.

Methods for extracting components from natural sources include drying, maceration [9], ultrasound-assisted extraction [10], microwave-assisted extraction [11], and superficial fluid extraction [12]. Drying is a simple, low-cost, and scalable extraction method that enables the efficient obtention of secondary metabolites by applying mild (20–60 °C) or elevated (>70 °C) temperatures [13,14], and freezing [15], oven [16], or vacuum drying [17]. Even though the variability in such experimental settings can influence the bioactivity and functional properties of the extracted substances, the importance of drying extraction has been reflected over the last decade in the obtention of compounds of highly valuable antitumoral [18], antidiabetic [19], antioxidant, and anti-inflammatory compounds [20].

Chicozapote fruits contain numerous biologically active compounds in their pulp, peels, and seeds [21]. Recent studies have demonstrated that the pulp and peels of chicozapote fruits are important sources of flavonoids or polyphenols such as methyl chlorogenate, dihydromyricetin, Qu, myricitrin, catechin, epicatechin, gallocatechin, and gallic acid [22]. In the same context, other studies have reported that the seeds of chicozapote fruits contain therapeutically attractive glycosidic derivatives of flavonoids such as D-quercitol and myricetin-3-*O*-*α*-L-rhamnoside [23]. Since these secondary metabolites possess the capacity to modulate molecular and cellular phenomena, preparations derived from chicozapote (e.g., extracts, juices, and syrups) have been useful to treat, traditionally, cases of burns [24], dysentery [25], wounds [26], pulmonary diseases (e.g., asthma, pulmonary fibrosis, and pneumonia) [27,28], cancer, and oxidative stress-related disorders such as Parkinson’s disease and cerebral ischemia [29].

Infectious diseases are caused by pathogenic microorganisms such as bacteria, viruses, fungi, or parasites [30]. According to the World Health Organization (WHO), bacteria that represent a threat to human health are categorized into critical (e.g., *Escherichia coli*, *Pseudomonas aeruginosa*, and *Klebsiella pneumoniae*), high (e.g., *Staphylococcus aureus*, *Helicobacter pylori*, and *Salmonellae*), and medium (e.g., *Streptococcus pneumoniae*, *Haemophilus influenzae*, and *Enterococcus faecalis*) priority [31].

Oxidative stress is a fundamental biological process that alludes to the imbalance between the production of free radicals and the capacity of organisms to neutralize or detoxify them. Various factors can promote harmful oxidative stress, for instance, overexposure to environmental factors, poor diet, unhealthy lifestyle, and aging [32,33]. The constant exposure to over-generated free radicals from oxidative stress results in the initiation and progression of a series of disorders that can decrease the functionality of the nervous, respiratory, cardiovascular, or metabolic systems [34,35]. Current approaches to reducing the consequences of oxidative stress are associated with the consumption of natural and synthetic alternatives such as fruits and supplements, respectively.

The cultivation and commercialization of *M. zapota* faces significant challenges despite its high nutritional value and traditional medicinal properties. The fruit is highly perishable, limiting its production and commercialization, with a maximum shelf life of only 11 days at room temperature. Chicozapote is valued for its sweet fruit and diverse bioactive compounds, including flavonoids, tannins, glycosides, and terpenoids. These compounds contribute to various biological activities, such as antioxidant, antimicrobial, anti-inflammatory, and antidiabetic effects. However, the extraction and preservation of these bioactive compounds pose significant challenges, necessitating a deeper understanding of the optimal extraction conditions, sample treatments, and drying methods.

This study sought to investigate, for the first time, the influence of drying methods (lyophilization and oven drying) on the phytochemical content and biological properties (i.e., antibacterial, antioxidant, and toxicity) of extracts from the pulp, peel, and seeds of chicozapote fruits, hypothesizing that the drying process significantly affects the yield of bioactive compounds extracted from chicozapote fruits, thereby affecting their biological activity. The general objective of this work was to evaluate the yield of bioactive compounds extracted from chicozapote fruit using different drying methods and distinct biological models. The total flavonoid content (TFC) and total phenol content (TPC) were analyzed for each extract. The antibacterial assays were conducted towards high and critical-priority Gram-positive and Gram-negative pathogens, whereas the antioxidant capacity was revealed using DPPH and ABTS radicals, respectively. The toxicity of the obtained extracts was studied against *Artemia salina*, which was appraised as an *in vivo* model. The identification of methyl ester derivatives among each extract was performed by GC-FID analyses. *In silico* analyses were implemented to investigate possible mechanisms of action regarding the antibacterial activity of chicozapote; this is in accordance with other reports where the presence of flavonoid derivatives has been reported. This work demonstrates for the first time the influence of drying methods in the antibacterial and antioxidant performance of extracts from chicozapote fruits and validates their activity through *in silico* and *in vivo* approaches.

## 2. Results

### 2.1. Bromatological Analyses

**Figure 1:**
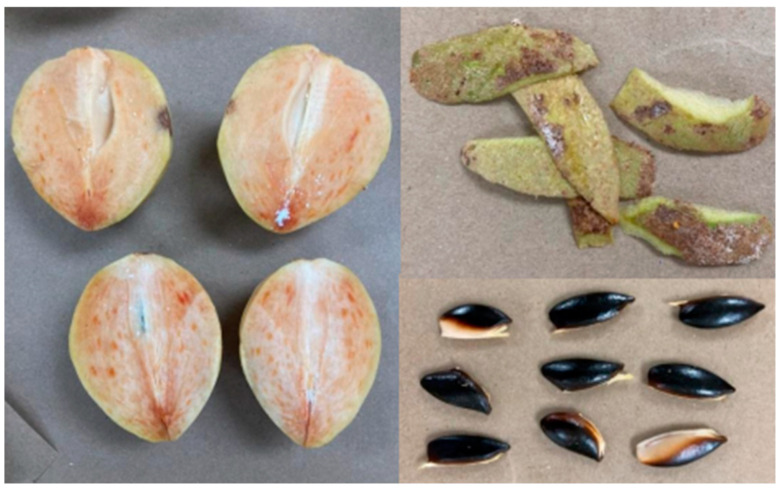
Representation of the collected different parts (peel, pulp, and seeds) of chicozapote fruits.

The initial bromatological analyses of the chicozapote fruits revealed that the moisture content of chicozapote fruits was 76.24%, whereas their pH, soluble solid content, and titratable acidity were 4.46, 19.51, and 0.56%, respectively. In the same regard, analyses demonstrated that the maturity index of fruit samples was 34.72. As noted in Figure 1, the fruit shape was round with a reddish pulp, scurfy peel, and shiny black seeds. The determined average weight, diameter, and length of chicozapote fruits were 169.22, 62.27, and 84.28 mm, respectively. The non-edible portion of the sample, including the peel and seeds, represented approximately 12.88% of the total weight, whereas the edible portion was 87.07%.

### 2.2. Color and Ripening Characteristics

**Figure 2:**
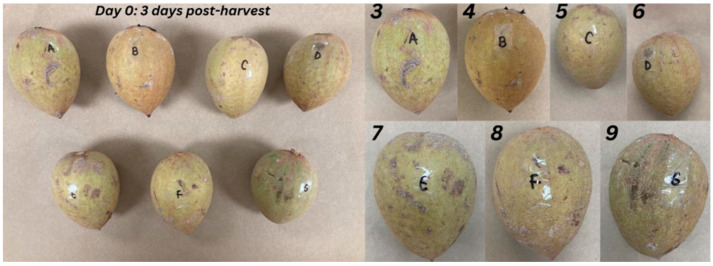
Ripening of chicozapote fruits after 9 days post-harvest under storage at room temperature. Numbers in the photographs represent the days of storage of different chicozapote fruits.

According to the International Commission on Illumination (CIE), the color parameters of the chicozapote pulp, peel, and puree were evaluated in terms of their lightness (L*), the position of the color on the red–green axis (a*), and the position of the color on the blue–yellow axis (b*). The calculated L* values for the pulp, puree, and peel were 55.77 ± 2.93, 58.28 ± 0.67, and 68.04 ± 3.10, respectively. On the other hand, the determined a* values for each sample were 11.28 ± 3.14, 14.87 ± 0.54, and 4.39 ± 1.56. The b* values of the peel, puree, and pulp were 23.19 ± 2.68, 38.81 ± 0.34, and 32.50 ± 2.4, respectively, and ranged from 23.19 to 32.50. The hue angle (*h_ab_*) of samples ranged from 0° to 360°. The ripening characteristics of the chicozapote fruits from 3 to 9 days post-harvest are illustrated in Figure 2.

According to Table 1, the moisture content of samples was reduced from 76.24 ± 2.11 to 68.2 ± 0.01%, approximately. Oppositely, it was recorded that the soluble solid contents of chicozapote fruits increased from 19.51 ± 0.5 to 21.91 ± 0.32 after nine days post-harvest. On the other hand, it was noted that chicozapote fruits at 3, 4, 5, and 6 days post-harvest exhibited 0.56 ± 0.04, 0.45 ± 0.09, 0.33 ± 0.03, and 0.21 ± 0.03% titratable acidity. The titratable acidity of samples also decreased after 7- (0.1 ± 0.003), 8- (0.095 ± 0.01), and 9 (0.09 ± 0.01) post-harvest days. Contrary to these results, the chicozapote maturity index rapidly increased during the evaluated period. It had an initial index of 34.72 ± 0.90, 35.45 ± 0.61, and 54.46 ± 2.80 at 3, 4, and 5 days post-harvest and reached 230.48 ± 3.45 by 6, 7, 8, and 9 days post-harvest, respectively.

### 2.3. Drying, Powdering, Proximate Composition, Extraction Yield, and Phytochemical Content of Chicozapote Fruits

#### 2.3.1. Drying and Powdering

The drying and powdering results for the different chicozapote samples are presented in Table 2. The yield (% *w*/*w*) of pulp after lyophilization and oven-dried methods was 26.12 ± 0.81 and 25.87 ± 0.34%, respectively. Comparably, the yield of lyophilized and oven-dried peel was 32.33 ± 1.18 and 32.68 ± 0.48. In contrast to pulp and peel samples, the use of lyophilization and oven-dry techniques occurred in higher yields: 54.06 ± 0.09 and 57.53 ± 2.49. Table 1 also contains the determined particle sizes of each sample. Figure S1 depicts visual differences between the processed samples by lyophilization and oven drying.

#### 2.3.2. Proximate Composition

The proximate composition of each section of the chicozapote fruit, which underwent lyophilization and oven-drying techniques, is presented in Table 3. As listed, the content of protein, ash, fat, insoluble dietary fiber (IDF), high-molecular-weight soluble dietary fiber (SDFP), total dietary fiber (TDF), and digestible carbohydrates (DC) varied in accordance with the implemented drying extraction method among pulp, peel, and seed samples. It can be noted that lyophilized peel (LPE) and seeds (LS) exhibited the highest protein content, occurring at 5.26 ± 0.59 and 5.25 ± 0.22% g/100 g dry sample (DS), respectively. Similarly, the lyophilization of pulp (LPU) occurred with a higher protein content (4.61 ± 0.60 g/100 g DS) than over-dried pulp (OPU) (4.34 ± 0.76 g/100 g DS).

Contrary to these results, OPU (7.91 ± 0.18 g/100 g DS) and OS (15.10 ± 2.25 g/100 g DS) exerted the highest ash content compared to LPU (7.62 ± 0.36 g/100 g DS) and LS (11.4 ± 2.70 g/100 g DS), respectively. Again, the OPU (0.93 ± 0.13 g/100 g DS) and OPE (6.43 ± 0.17 g/100 g DS) were higher than the LPU (0.78 ± 0.06 g/100 g DS) and LPE (0.71 ± 0.01 g/100 g DS) samples, respectively. The fat content of LS and OS was 16.09 ± 0.38 and 10.48 ± 0.30 g/100 g DS, respectively. The IDF content was higher among lyophilized samples; for instance, LPU and LPE presented 21.8 ± 1.50 and 29.6 ± 0.08 g/100 g DS, whereas LS resulted in 59.58 ± 3.56 g/100 g DS, respectively. Regarding the analysis of OPU, OPE, and OS samples, it was determined that their IDF content was 17.88 ± 0.54, 23.53 ± 0.72, and 55.02 ± 8.90 g/100 g DS, respectively. The recorded SDFP content of LPU, LPE, and LS was 19.50 ± 1.40, 22.32 ± 0.97, and 22.32 ± 0.97 g/100 g DS, respectively. In contrast, the assessed SDFP content of OPU, OPE, and OS was 13.74 ± 1.03, 11.51 ± 1.74, and 3.56 ± 0.35 g/100 g DS. The TDF content of LPU, LPE, and LS was higher compared to OPU (31.61 ± 1.56 g/100 g DS), OPE (35.04 ± 1.73 g/100 g DS), and OS (58.57 ± 3.25 g/100 g DS), resulting in 41.37 ± 3.03, 51.92 ± 1.04, and 64.88 ± 3.83 g/100 g DS, respectively. The DC content of LPU, LPE, and LS was slightly low compared to oven-dried samples since OPU, OPE, and OS occurred in 59.20 ± 3.63, 45.92 ± 5.72, and 12.02 ± 0.59 g/100 g DS, respectively.

#### 2.3.3. Extraction Yield and Phytochemical Content

**Figure 3:**
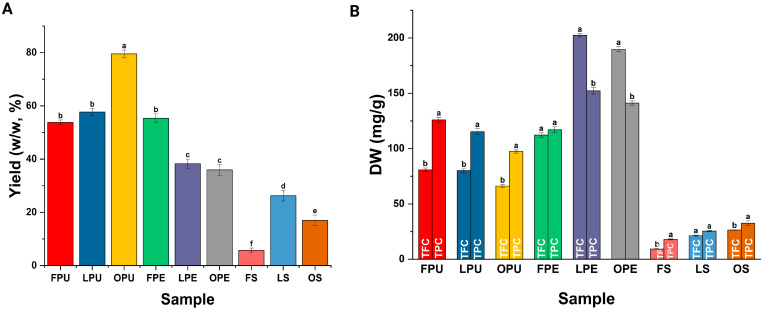
(**A**) Extraction yield and (**B**) TFC and TPC of lyophilized and oven-dried pulp, peel, and seeds from chicozapote fruits. Values are presented as the mean ± SD. FPU, fresh pulp; LPU, lyophilized pulp; OPU, oven-dried pulp; FPE, fresh peel; LPE, lyophilized peel; OPE, oven-dried peel; FS, fresh seed; LS, lyophilized seed; OS, oven-dried seed. Letters a–f represent statistical differences between results.

As illustrated in Figure 3A, the extraction yields of bioactive compounds from LPU and OPU samples were 57.7 ± 1.33 and 79.5 ± 1.45%, whereas the extraction yields of LPE, OPE, LS, and OS were 38.26 ± 1.74, 35.93 ± 1.99, 26.24 ± 2.01, and 17.01 ± 1.97% respectively. As depicted in Figure 3B, the recorded TFCs of fresh pulp (FPU), LPU, and OPU samples were 80.76 ± 1.7, 80.19 ± 1.67, and 66.15 ± 1.4 quercetin equivalents per gram of extract (mg QuE/g), respectively. The determined TPC of the same samples was 125.87 ± 2.28, 115.19 ± 2.67, and 97.34 ± 1.44 gallic acid equivalents per gram of extract (mg GAE/g), respectively. On the other hand, the calculated TFC of fresh peel extract (FPE), LPE, and OPE samples was 189.71 ± 2.25, 202.37 ± 1.50, and 112.28 ± 20 (mg QuE/g), whereas their TCT was 216.34 ± 2.51, 242.43 ± 0.53, and 244.27 ± 3.47, respectively. The TPC of FPE, LPE, and OPE was 141.14 ± 1.97, 152.15 ± 2.88, and 117.01 ± 2.63 mg GAE/g, respectively. Regarding the composition of FS, LS, and OS samples, it was recorded that their TFC was 9.30 ± 0.26, 21.36 ± 0.57, and 26.93 ± 0.25 mg QuE/g. The TPC of the samples was 17.91 ± 0.43, 25.48 ± 0.48, and 32.68 ± 2.01 mg GAE/g, respectively. The calibration curves constructed to calculate the TPC, TFC, and TCT content of chicozapote samples are illustrated in the Supplementary Materials (Figures S1 and S2).

### 2.4. GC-FID Analysis of Methyl Esters

According to Table 4, GC-FID analysis revealed the presence of different methyl ester derivatives of fatty acids from chicozapote samples. Chromatograms from GC-FID analyses are presented in Supplementary Materials (Figures S3–S11).

### 2.5. Antibacterial Activity

The antibacterial activity of lyophilized and oven-dried pulp, peel, and seeds from chicozapote was tested against a panel of Gram-positive (*S. aureus*, and *E. faecalis*), and Gram-negative (*E. coli*, *P. aeruginosa*, *S. enterica* spp. *arizonae*, and *K. pneumoniae*). As presented in Table 5, FPU, LPU, and OPU inhibited the growth of *E. coli* at 800 μg/mL, whereas FPE, LPE, and OPE exerted their activity at 600, 396, and 1000 μg/mL, respectively. Against the same strain, it was observed that treatment with FS, LS, and OS was ineffective, as the MIC for each sample was determined at >1000 μg/mL. Similarly, the FPU, LPU, and OPU inhibited the growth of *S. aureus* at 1000 μg/mL, whereas FPE, LPE, and OPE inhibited its growth at 396 and 800 μg/mL. Again, FS, LS, and OS performed the lowest activity against *S. aureus* at >1000 μg/mL. Towards *S. enterica* spp. *arizonae*, it was noted that treatment with FPU and LPU inhibit its growth at 800 μg/mL, whereas treatment with OPU was effective at 1000 μg/mL. Treatment with FPE, LPE, and OPE sample peels occurred in similar MIC values: 396 and 800 μg/mL. The activity of FS, LS, and OS against *S. enterica* spp. *arizonae* was observed at >1000 μg/mL. The MIC of FS, LS, and OS against *P. aeruginosa* was recorded at >1000 μg/mL, which was similar to FPU, LPU, and OPU. The capacity of FPE, LPE, and OPE to inhibit the growth of *P. aeruginosa* was observed at 396, 198, and 1000 μg/mL, respectively. Comparably to these findings, the activity of lyophilized and oven-dried pulp and seeds was noted at >1000 μg/mL against *K. pneumoniae* and *E. faecalis*. Towards the former, FPE, LPE, and OPE inhibit its growth at 396, 198, and 1000 μg/mL, whereas against the latter, they were active at 198 and 1000 μg/mL.

### 2.6. Analysis of Antibacterial Activity In Silico

Before performing the molecular docking of the metabolites on DNA gyrase B, the computational parameters were validated using the self-docking method, which employed structures that had been co-crystallized with an inhibitor. Root mean square deviation (RMSD) values less than 2 Å were obtained in all cases. Specifically, for methyl-4-hydroxycinnamate from 7DPS, a value of 0.04673 Å was obtained; for the inhibitor ULD-2 (PubChem CID: 151595514) of 1.5445 Å in 6TCK, 0.8685 Å for DOO (PubChem CID: 66560858) on 4K4O, and finally, the self-docking calculation for R53 (PubChem CID: 167530343) gave a value of RMSD = 1.2476 Å on the crystal 8BN6. Table 6 describes the energy values obtained for each metabolite on the DNA Gyrase B of the bacterial species under investigation.

**Figure 4:**
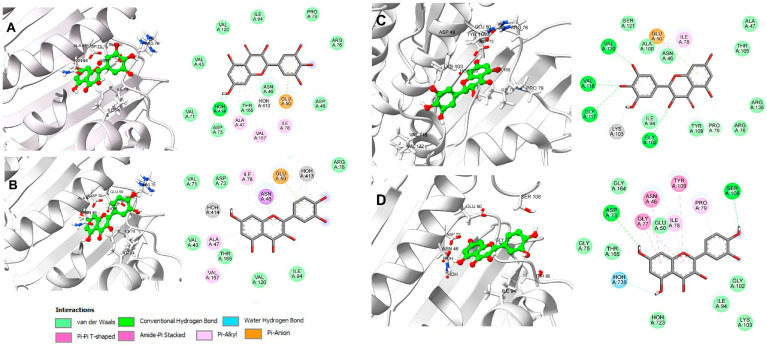
Binding mode and 2D diagram of molecular interactions on DNA gyrase: (**A**) dihydromyricetin (−7.0 kcal/mol) and (**B**) Qu (−6.6 kcal/mol) on *E. coli* (PDB ID: 7DPS); (**C**) dihydromyricetin (−8.8 kcal/mol) and (**D**) Qu (8.4 kcal/mol) on *S. enterica* (PDB ID: 8J9T).

As listed in Table 6, dihydromyricetin and Qu obtained an average affinity of −8.2 kcal/mol and −8.1 kcal/mol, respectively, indicating a favorable affinity for the catalytic cavity of gyrase B. Figure 4 illustrates the molecular recognition of these two metabolites on the ATP binding site, as well as the diagram of molecular interactions that determine the ligand–protein binding on the DNA gyrase of *E. coli* and *S. enterica* strains that were susceptible to treatment with FPU, LPU, OPU, FPE, and LPE, respectively.

The interactions generated by dihydromyricetin and Qu and the ATP recognition site in *E. coli* were predominantly of the pi-alkyl type, involving the amino acids Ala47, Ile78, and Val167. Additionally, there were pi-anion interactions with Glu50 and hydrophobic interactions with the residues Val43, Val71, Ile94, and Val120 (Figure 4A,B). Regarding the binding mode of dihydromyricetin on *S. enterica* 8J9T, the formation of hydrogen bond interactions with Gly102, Gly117, Val118, and Val120, a pi-anion interaction with Glu50, a pi-alkyl interaction with Ile78, and the remainder with hydrophobic amino acids such as Ala47, Pro79, Ile94, and Ala100 can be observed (see Figure 4C). While Qu maintained a hydrogen bond interaction with Asp73 and Ser108, it also exhibited pi-pi T-shaped interactions with Asn46, Gly77, and Tyr109, a pi-alkyl interaction with Ile78 and Pro79, and finally, hydrophobic interactions with Ile94 (see Figure 4D). The metabolites in order of affinity energy obtained are chlorogenic acid and myricitrin. They are of biological relevance due to their presence in extracts with antibacterial activity.

**Figure 5:**
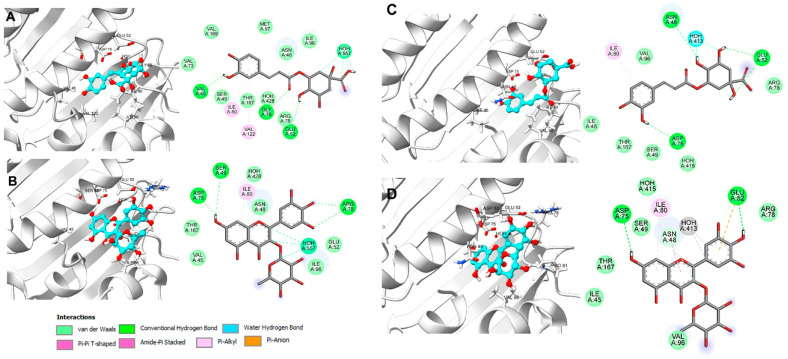
Binding mode and 2D diagram of molecular interactions on DNA gyrase: (**A**) chlorogenic acid (−8.0 kcal/mol) and (**B**) myricitrin (−7.9 kcal/mol) on *P. aureginosa* (PDB ID: 8BN6); (**C**) chlorogenic acid (−8.2 kcal/mol) and (**D**) myricitrin (−8.3 kcal/mol) on *E. faecalis* (PDB ID: 4K4O).

When analyzed on the DNA gyrase of different bacterial species, the average affinity energy of the two metabolites was −7.9 kcal/mol. Figure 5 illustrates the binding mode of these metabolites on the ATP binding site in gyrase B of *P. aureginosa* and *E. faecalis*. Chlorogenic acid showed a binding free energy of −8.0 kcal/mol over 8BN6, determined by hydrogen bond interactions with the amino acids Val45, Glu52, Gly79, and HOH557, pi-alkyl interactions with Ile80 and Val122, as well as van der Waals interactions with Val73, Ile96, Met97, and Val196, mainly (Figure 5A). Similarly, myricitrin adopted a binding mode analogous to that of novobiocin and chlorogenic acid, reaching an affinity energy of 7.9 kcal/mol through hydrogen bond interactions with Ser49, Asp75, Arg78, and HOH557 (Figure 5B) while maintaining the pi-alkyl and hydrophobic interactions observed in the previous case. In contrast, chlorogenic acid demonstrated a binding energy of 8.2 kcal/mol with *E. faecalis* DNA gyrase, indicating that molecular recognition occurs through hydrogen bond interactions with Asn48, Glu52, and HOH413 (Figure 5C). Considering the same protein, myricitrin adopted a binding mode determined by hydrogen bond interactions with Glu52 and Asp75 and pi-alkyl interactions with Ile80, and hydrophobic interactions with Ile45, Val96, and Pro81. This resulted in an energy value of −8.3 kcal/mol (Figure 5D).

### 2.7. Analysis of Antioxidant Activity

**Figure 6:**
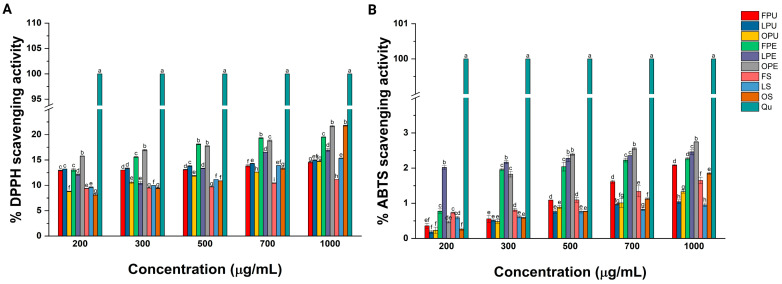
Antioxidant capacity of lyophilized and oven-dried chicozapote extracts following the (**A**) DPPH and (**B**) ABTS assays. Values are presented as the mean ± SD. FPU, fresh pulp; LPU, lyophilized pulp; OPU, oven-dried pulp; FPE, fresh peel; LPE, lyophilized peel; OPE, oven-dried peel; FS, fresh seed; LS, lyophilized seed; OS, oven-dried seed; Qu, quercetin. Letters a–g represent statistical differences between results.

The antioxidant activity of chicozapote samples was assessed via the DPPH and ABTS assays, respectively. As illustrated in Figure 6A, treatment with 200, 300, and 500 μg/mL of FPU scavenged 12.91 ± 0.08 and 13.00 ± 0.01% DPPH radicals, whereas treatment at 700 and 1000 μg/mL inhibited 13.83 ± 0.23 and 14.52 ± 0.11% free radicals, respectively. The treatment with 200 and 300 μg/mL of LPU inhibited 13.21 ± 0.02 and 13.34 ± 0.10% DPPH radicals. At 500, 700, and 100 μg/mL, treatment with LPU scavenged 13.82 ± 0.12, 14.27 ± 0.05, and 14.99 ± 0.08% DPPH radicals, respectively. Similarly, treatment with 200 and 300 μg/mL of OPU scavenged 8.80 ± 0.07 and 10.58 ± 0.25, whereas at 500 and 700 μg/mL, treatment with OPU inhibited 11.86 ± 0.12 and 12.59 ± 0.02% DPPH free radicals. At 1000 μg/mL, it was observed that treatment with OPU scavenged 14.73 ± 0.05% DPPH radicals.

As noted in Figure 6A, treatment with FPE at 200 and 300 μg/mL inhibited 13.05 ± 0.28 and 15.58 ± 0.10% DPPH radicals, whereas treatment at 500, 700, and 1000 μg/mL scavenged 18.11 ± 0.10, 19.33 ± 0.09, and 19.50 ± 0.05% free radicals, respectively. Comparably, treatment with 200, 300, and 500 μg/mL of LPE scavenged 10.44 ± 0.33, 12.12 ± 0.14, and 13.34 ± 0.05% DPPH radicals, respectively. At 700 and 1000 μg/mL, treatment with LPE occurred in 16.54 ± 0.01 and 16.95 ± 0.36% inhibited free radicals. The treatment with 200 and 300 μg/mL of OPE caused the scavenging of 15.76 ± 0.05 and 16.94 ± 0.16, whereas at 500 and 700 μg/mL, it inhibited the formation of 17.76 ± 0.05 and 18.82 ± 0.14% DPPH radicals, respectively. Treatment with 1000 μg/mL of OPE had the highest antioxidant performance since it inhibited 21.67 ± 0.15% DPPH radicals.

As observed in Figure 6A, treatment with 200, 300, and 500 μg/mL of FS inhibited the generation of 9.37 ± 0.03, 9.47 ± 0.02, and 9.67 ± 0.02% DPPH radicals, respectively. Similarly, treatment with 700 and 1000 μg/mL of FS scavenged 10.45 ± 0.05 and 11.17 ± 0.07% radicals. The treatment with 200 and 300 μg/mL of LS occurred in the inhibition of 9.64 ± 0.09 and 9.96 ± 0.02% DPPH radicals, whereas treatment with 500, 700, and 1000 μg/mL inhibited 11.16 ± 0.01, 13.90 ± 0.01, and 15.33 ± 0.10% free radicals, respectively. Contrarily, treatment with 200 and 300 μg/mL of OS resulted in the scavenging of 8.13 ± 0.16 and 9.42 ± 0.10% DPPH radicals, whereas at 500 and 700 μg/mL, treatment occurred in 10.88 ± 0.02 and 13.32 ± 0.24% inhibited free radicals, respectively. The highest antioxidant activity for OS was recorded at 1000 μg/mL, as it occurred in 21.76 ± 0.10% scavenged radicals. Qu was utilized as the positive control, and initial treatment occurred in 100% scavenged DPPH radicals.

As depicted in Figure 6B, treatment with 200 and 300 μg/mL of FPU scavenged 0.35 ± 0.07 and 0.55 ± 0.09% ABTS radicals, whereas at 500, 700, and 1000 μg/mL, it inhibited 1.08 ± 0.05, 1.61 ± 0.04, and 2.08 ± 0.02% free radicals, respectively. Comparably, treatment with 200, 300, and 500 μg/mL of LPU scavenged 0.18 ± 0.04, 0.51 ± 0.02, and 0.76 ± 0.04% ABTS radicals. At 700 and 1000 μg/mL, treatment with LPU scavenged 0.96 ± 0.01 and 1.04 ± 0.05% free radicals, respectively. The treatment with 200 and 300 μg/mL of OPU inhibited the formation of 0.23 ± 0.08 and 0.48 ± 0.05% ABTS radicals, whereas at 500 and 700 μg/mL, it occurred in the scavenging of 0.89 ± 0.05 and 1.01 ± 0.12% free radicals, respectively. The treatment with 1000 μg/mL of OPU scavenged 1.33 ± 0.04% ABTS radicals.

As represented in Figure 6B, treatment with 200, 300, and 500 μg/mL of FPE inhibited 0.77 ± 0.06, 1.95 ± 0.03, and 2.04 ± 0.12% ABTS radicals, respectively. At 700 and 1000 μg/mL, treatment with FPE occurred in the inhibition of 2.22 ± 0.05 and 2.26 ± 0.03% ABTS radicals. Comparably, treatment with 200 and 300 μg/mL of LPE scavenged 2.02 ± 0.05 and 2.17 ± 0.04% ABTS radicals, whereas at 500 and 700 μg/mL, it inhibited 2.27 ± 0.09 and 2.35 ± 0.05% free radicals, respectively. At 1000 μg/mL, treatment with LPE inhibited 2.46 ± 0.06% free radicals. The treatment with 200, 300, and 500 μg/mL of OPE scavenged 0.47 ± 0.02, 1.82 ± 0.07, and 2.39 ± 0.03% ABTS radicals. Similarly, treatment at 700 and 1000 μg/mL of OPE inhibited 2.55 ± 0.03 and 2.74 ± 0.05% ABTS radicals.

As represented in Figure 6B, treatment with 200 μg/mL of FS scavenged 0.73 ± 0.01% ABTS radicals, whereas at 300, 500, and 700 μg/mL, it inhibited the generation of 0.80 ± 0.04, 1.09 ± 0.06, and 1.34 ± 0.15% free radicals, respectively. At 1000 μg/mL, treatment with FS scavenged 1.65 ± 0.08% ABTS radicals. Regarding the activity of LS, it was determined that treatment at 200 and 300 μg/mL scavenged 0.59 ± 0.03 and 0.061 ± 0.02% free radicals, whereas at 500 and 700 μg/mL, it inhibited 0.76 ± 0.04 and 0.83 ± 0.04% ABTS radicals, respectively. At 1000 μg/mL, the treatment with LS scavenged 0.95 ± 0.05% ABTS radicals. Similarly, the treatment with 200, 300, and 500 μg/mL of OS inhibited 0.26 ± 0.03, 0.58 ± 0.01, and 0.77 ± 0.05% ABTS radicals, respectively. At 700 and 1000 μg/mL, treatment with OS caused the scavenging of 1.12 ± 0.02 and 1.85 ± 0.03% ABTS radicals. Qu was also utilized as the positive control, and treatment with 200–1000 μg/mL inhibited the formation of 100% ABTS radicals.

According to their capacity to scavenge DPPH and ABTS radicals, Table 7 presents the half-maximal inhibitory concentration (IC_50_) of each sample.

### 2.8. Analysis of In Vivo Toxicity

**Figure 7:**
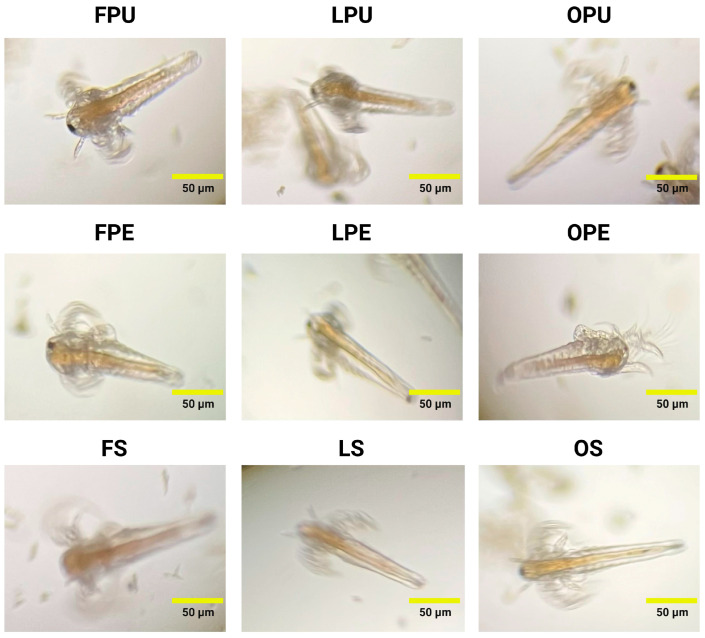
Representative images of the toxicity evaluation of lyophilized and oven-dried chicozapote fruits at 1000 μg/mL. FPU, fresh pulp; LPU, lyophilized pulp; OPU, oven-dried pulp; FPE, fresh peel; LPE, lyophilized peel; OPE, oven-dried peel; FS, fresh seed; LS, lyophilized seed; OS, oven-dried seed.

The *in vivo* toxicity of chicozapote samples was studied against *A. salina* nauplii. As observed in Figure 7, the treatment with FPU, LPU, or OPU at the proposed concentrations in this study (200, 300, 500, 700, and 1000 μg/mL) did not compromise the survival rate of *A. salina* nauplii nor caused significant anatomical alterations after 24 h exposure to treatment. Comparable effects were observed when FPE, LPE, OPE, FS, LS, and OS samples were tested.

## 3. Discussion

Traditional medicine includes cultural practices, beliefs, and knowledge of communities using plant-based remedies, acupuncture, and spiritual healing to restore the balance of body, mind, and spirit. In traditional medicine, preparations (e.g., juices, powders, or extracts) from fruits (e.g., pomegranate, papaya, or berries) have been utilized to develop nutritional supplements, detoxification regimens, tonics, and remedies for treating infectious, carcinogenic, metabolic, and neurodegenerative disorders. Chicozapote has been used traditionally by various civilizations because of its digestive, nutritional, antioxidant, and wound-healing benefits. In this work, chicozapote extracts were obtained by two distinct extraction techniques and further evaluated for their bromatological features, phytochemical content, and biological performance.

Extracts are concentrated substances derived from organic materials obtained by extraction methods, which can be classified according to the utilized solved, physical principles involved, and targeted compounds. Lyophilization, also known as freeze drying, is a preservation process that enables the extraction of bioactive compounds from fruits by including sublimation and desorption processes. Comparably, the oven-drying approach also allows the extraction of substances through heat application, resulting in moisture removal and concentration of compounds. Here, it was recorded that lyophilization or oven drying did not affect the extraction yield (25.87–57.53% *w*/*w*) nor particle size (0.074–0.40) from the pulp, peel, or seeds of chicozapote fruits. Regarding proximate composition analyses, it was found that the use of different extraction techniques significantly influenced the content of IDF (21.80 ± 1.50 and 17.88 ± 0.54), SDFP (19.50 ± 1.40 and 13.74 ± 1.03), and TDF (41.37 ± 3.03 and 31.61 ± 1.56) among LPU and OPU samples. Comparably, it was noted that the oven-drying approach resulted in a higher content of fat (6.43 ± 0.17) and DC (45.92 ± 5.72) in OPE. In contrast, lyophilization increased IDF (29.6 ± 0.08), SDFP (22.32 ± 0.97), and TDF (51.92 ± 1.04) content in LPE. Similar effects were observed among LS and OS samples regarding their fat (16.09 ± 0.38 and 10.48 ± 0.30), SDFP (5.31 ± 0.27 and 3.56 ± 0.35), TDF (64.88 ± 3.83 and 58.57 ± 3.25), and DC (7.12 ± 0.12 and 12.02 ± 0.59) content. The determination of water, protein, ash, IDF, TDF, or SDFP was not determined for fresh chicozapote samples since their heterogeneous nutrient distribution and high moisture content can hamper the analysis of such parameters. On the other hand, it was noted the yield of lyophilized extract from the pulp remained similar than its fresh counterpart. This can be due to the fact that when fruits are dried, the soluble compounds become more concentrated. The drying process typically preserves these compounds, leading to a yield that, when expressed as a percentage of the original fresh weight, can appear similar. On the other hand, this effect can be related to the fact that certain bioactive compounds, (e.g., polyphenols and vitamins), may be stable during the drying process. If these compounds are retained in similar amounts, the yield of the dried extract may closely match that of the fresh counterpart.

The quantification of potentially bioactive compounds among fruits is necessary to contribute to understanding their health outcomes and play a further role in developing therapeutic strategies against clinical challenges. Here, the TPC, TFC, and TCT of chicozapote samples were calculated by considering the capability of their phytoconstituents to react with specific chemical reagents such as the Folin–Ciocalteu reagent, aluminum chloride (AlCl_3_), and hydrochloric acid (HCl) and vanillin. The TPC assay is based on the capacity of hydroxyl groups from phenolic compounds to reduce molybdenum ions, occurring in the formation of a hetero-polyblue complex, which can be quantified by UV–vis spectrophotometry approaches [38]. Comparably, the TFC is correlated with the capability of the hydroxyl groups of flavonoids to form complexes with aluminum ions due to free electron pairs [39]. On the other hand, the TCT is related to the reaction between the hydroxyl groups from condensed tannins and vanillin, which is catalyzed by the presence of HCl and results in the formation of pink-colored complexes [40].

According to the retrieved equation (*y* = 0.0035*x* + 0.0029; *R*^2^ = 0.9704) from the calibration curve (see Figure S1), and as illustrated in Figure 3B, the TPC of chicozapote extracts was significantly different (*p* < 0.05) between the lyophilized and oven-dried peel and seed than their fresh counterparts. However, this was not observed for FPU, LPU, and OPU samples. In the same context, it was noted that the TFC of fresh, lyophilized, and oven-dried extracts from the seeds was significantly lower (*p* < 0.05) than the TFC of lyophilized and oven-dried extracts from the pulp and peel of chicozapote. Still, no statistical differences in the TFC between lyophilized and oven-dried extracts from the pulp and peel were determined. This is considering the regression equation retrieved from the calibration curve presented in Figure S2: *y* = 0.0007*x* + 0.069; *R*^2^ = 0.9877. It is noteworthy to mention that the TPC of dried extracts was lower since during the drying process some of the phenolic compounds might be degraded or transformed into their less active forms. In the same context, factors such as exposure to oxygen, alteration in the fruit matrix, and loss of volatile compounds might also contribute to the retrieved result. Similarly, such factors can affect the CaE content of the obtained lyophilized and oven-dried extracts from the pulp, peel, and seeds of chicozapote.

Chromatography is a frequently applied analytical approach for separating and analyzing components in a complex mixture such as extracts. There are different chromatography approaches (e.g., liquid, column, and ion chromatography) to investigate the phytoconstituents of extracts, and they are selected based on the sample’s polarity, thermal stability, and complexity. GC-FID is a highly versatile and sensitive technique to identify natural products based on vaporizing volatile and semi-volatile compounds, which are further carried by an inert gas and interact with a defined stationary phase. Contrarily to methods coupled to mass spectrometers, GC-FID provides basic information about natural products based on their retention times, which can be matched to known standards to identify the chemical composition of extracts tentatively. As listed in Table 4, lyophilization and oven drying influenced the abundance of methyl ester derivatives among the obtained samples. It was observed that myristic and pentadecanoic acids were only identified in FS (0.19 and 0.09%), LS (0.21 and 0.09%), and OS (0.21 and 0.10%). Contrarily, fatty acids such as palmitic, heptadecanoic, stearic, oleic, linoleic, arachidic, and linolenic acid were identified in all the obtained samples from the pulp, peel, and seed of chicozapote fruits.

Infectious diseases are caused by pathogenic microorganisms such as bacteria, fungi, viruses, and parasites. In human health care settings, bacterial infections are problematic due to their highly dynamic transmission, capacity to infect immunocompromised patients [41], surface persistence [42], and capability to evade the activity of current approaches due to the drug resistance mechanisms such as enzymatic degradation, alteration of target sites, and overexpression of efflux pumps [43]. Depending on their structural features, bacteria are categorized into Gram-positive and Gram-negative bacteria. Among Gram-negative bacteria, *E. coli* is predominantly related to foodborne diseases such as diarrhea [44], whereas *P. aeruginosa* and *K. pneumoniae* are frequently associated with pneumonia and skin, soft tissue, and bloodstream infections [45]. Similarly, the incidence of *S. enterica* spp. *arizonae* has been correlated to DNA damage in the intestinal mucosa *in vivo* [46]. On the other hand, the high prevalence of Gram-positive strains such as *S. aureus* in healthcare settings is correlated to catheter-related bloodstream infections, toxic shock syndrome, and invasive infections such as endocarditis, meningitis, and bacteremia [47]. In the same context, the incidence of *E. faecalis* has been documented to cause cystitis and pyelonephritis together with intra-abdominal and pelvic infections [48].

In this work, it was recorded that the treatment with lyophilized extracts obtained from the pulp and peel from chicozapote inhibited the growth of *E. coli* at 396–800 μg/mL. This effect was not observed for the lyophilized extract from the seed, which was active at >1000 μg/mL. In the same sense, the oven-dried extract from the pulp exerted similar activity against *E. coli* at 800 μg/mL; however, the oven-dried extract from the peel and seeds was effective at >1000 μg/mL. Against *S. enterica* spp. arizonae, another Gram-negative strain noted that treatment with extracts exhibited similar patterns, with the FPE and LPE extracts being the most active ones (396 μg/mL) and the oven-dried extracts the weakest (>1000 μg/mL). Treatment with FPE and LPE was also effective against *P. aeruginosa* at 396 and 198 μg/mL, respectively. Among the obtained extracts, the superior activity of LPE can be associated with its palmitic acid, linoleic acid, and linolenic acid content, which are fatty acids widely recognized because of their antibacterial performance [49,50,51].

Despite the activity of extracts and following the classification from the Clinical and Laboratory Standards Institute (CLSI) guidelines about antibiotic susceptibility, the antibacterial activity of the obtained extracts can be appraised as ineffective. The variability regarding the antibacterial activity of these extracts can be due to the applied drying method, which may influence the content of bioactive compounds. In addition, it can be associated with their limited solubility, poor viability, and narrow target specificity, which are common limitations regarding the use of fruit-based therapeutic strategies that require additional efforts to improve their bioactivity.

*In silico* analyses are reliable computer-based simulations to predict synthetic or natural compounds’ biological and chemical features as they can efficiently model their interaction with molecular or cellular components. Compared to *in vitro* assays, *in silico* approaches are preferred due to their cost effectiveness, integration with experimental data, and importance in elucidating possible novel mechanisms of action [52,53]. The metabolites evaluated by molecular docking can be classified into phenolic (catechin, chlorogenic acid, methyl chlorogenate, syringic acid, and vanillic acid), flavonoid derivatives (dihydromyricetin, myricitrin, and Qu), and carotenoid (lutein) compounds. Phenolic compounds and flavonoids have shown antibacterial activity by inhibiting the activity of DNA gyrase B, an enzyme essential for bacterial replication, which relieves the tension within double-stranded DNA during DNA replication, causing cell death. In contrast, carotenoids such as lutein have been shown to possess antibacterial activity, exerting an inhibitory effect on biofilm formation and quorum sensing. Considering the mechanism of action of lutein, the low affinity and poor molecular recognition for the ATP binding site in DNA gyrase can be explained, according to our data. Particularly, polyphenolic derivatives have been reported to have an affinity and activity with DNA gyrase, such as gallic acid, catechin, and chlorogenic acid.

Chemically, their inhibitory capacity has been attributed to their ability to generate hydrogen bond interactions with hydroxyl groups distributed over the aromatic ring and critical residues in ATP recognition, such as Asp, Asn, Gly, and Thr, interactions that we were able to identify in our *in silico* analyses. However, flavonoids such as dihydromyricetin, myricitrin, and Qu have been carefully characterized as DNA gyrase inhibitors and housed in the ATP binding cavity. Generally, flavonoids with methoxylated and glycosylated substituents exhibit reduced activity compared to those with free hydroxyl groups. This may explain the superior affinity energy of dihydromyricetin and Qu over myricitrin. The above results corroborate the suitability of the calculated parameters for the reproducibility of theoretical conformations of the inhibitors since they are analogous to the bioactive conformations. The conformational alignment of each inhibitor is presented in Figures S11 and S12. The affinity energies obtained from the docking analysis indicated that the most favorable metabolites were catechin, chlorogenic acid, dihydromyricetin, myricitrin, and Qu, with an average energy of −8.0 kcal/mol. This value was slightly lower than that obtained for novobiocin (−8.2 kcal/mol).

Frequent methods to study the antioxidant activity of extracts include the DPPH and ABTS assays. The DPPH and ABTS assays are colorimetric methods based on the ability of compounds to neutralize their formation by prevention or chain-breaking mechanisms. Both assays are commonly considered for investigating potential therapeutic extracts because of their simplicity, ease of use, reproducibility, and cost effectiveness. Here, it was observed that the capacity of extracts from the pulp, peel, or seeds of chicozapote obtained via lyophilization and oven drying is poor due to their IC_50_ values (>2000 μg/mL). The data retrieved from both assays are challenging to compare since few studies have reported the antioxidant performance of extracts from chicozapote, and they are expressed as Trolox or vitamin C equivalent antioxidant capacity or are related to drying temperature (25–100 °C) [54]. Another parameter that influences such results is the amount of raw material utilized for extract preparation, where, in other studies, it has been ~5 kg [55]. In the DPPH assay, samples with higher DPPH scavenging activity included oven-dried extracts from the peel and seeds of chicozapote, followed by the fresh extract of the peel. On the other hand, samples with the highest ABTS scavenging activity include the lyophilized extracts from the peel together with the oven-dried counterparts. Again, fresh extract from the peel exerted the highest activity. The observations were made because the use of distinct drying methods favored the extraction of compounds that were not identified in this study but influenced the antioxidant capacity of the mentioned extracts. The superior activity of dried extracts from chicozapote can be associated with fresh sample extracts still containing a small amount of water, which might influence its concentration of secondary metabolites and compromise their antioxidant performance.

Toxicity assays are required to evaluate the potential risks associated with the exposure and environmental impact of substances. The toxicity of extracts can be evaluated through *in vitro* and *in vivo* methods. *A. salina* belongs to the genus *Artemia*, a broad genus of crustaceans found in saltwater environments. Contrary to other commercially available models, *A. salina* nauplii are easily cultivated under controlled saline conditions and exhibit a short life cycle, enabling their quick reproduction and growth for research studies. Here, *A. salina* nauplii were cultured to analyze the potential toxicity of lyophilized and oven-dried chicozapote extracts at the proposed concentrations (200, 300, 500, 700, and 1000 μg/mL) after 24 h exposure. Even though no significant changes in the anatomy of nauplii were observed, treatment with potassium dichromate (K_2_Cr_2_O_7_) occurred in the viability of 10% *A. salina* nauplii; this effect was statistically different (*p* < 0.05) from treatment with the obtained lyophilized and oven-dried extracts. The retrieved results are challenging to compare since this is the first time an *in vivo* model has been utilized to compare the toxicity of extracts from chicozapote obtained from distinct drying methods. Despite their scarce toxicity, the observed effect of extracts is also complex to compare with international guidelines since current approaches, such as the ISO/TS 20787:2017 [56], guideline, are focused on determining the aquatic toxicity of nanomaterials, structures with nanometric ranges that exert different physical, chemical, and biological behavior than their macroscopic counterparts due to their size, shape, and surface charge. Additional efforts are required to validate and establish parameters that assess the toxicity effects of natural-based preparations.

## 4. Materials and Methods

### 4.1. Reagents

Gallic acid, quercetin, ascorbic acid, (+)-catechin, FC reagent, sodium carbonate, aluminum trichloride, sodium nitrite, sodium hydroxide, hydrochloric acid, vanillin, 2,2-Diphenyl-1-picrylhydrazyl (DPPH), 2,2′-azino-bis(3-ethylbenzothiazoline-6-sulfonic acid (ABTS), and reagents needed for GC-FID analyses were analytical grade and purchased from Sigma Aldrich (Steinheim, Germany). *A. salina* cysts and artificial sea salt were obtained from a commercial supplier in San Andrés Cholula, Puebla, México.

### 4.2. Obtention and Processing of Plant Material

Chicozapote fruits were obtained commercially from Uruapan, Michoacan, Mexico. Harvesting occurred in October 2022, coinciding with the peak season of the fruit in this region. Furthermore, they were identified by the biologist Juan Jose Montiel-Avila at Vivero Maculxóchitl (La Purificación, Texcoco, Estado de México, México); specimens were deposited with the voucher number 0211ASL. Once collected, fruits were processed, washed, and disinfected after a ripening period of three days. Then, the fruits were divided into sections, and the peel and seeds were carefully removed. The resulting pulp was processed into a puree by using a food homogenizer, packed in plastic bags, and protected from light.

Freeze drying was conducted through lyophilization using Labconco 7,400,040 lyophilizer (Kansas City, MO, USA). After storage at −80 °C for at least three days, the frozen sections were ready for processing. The freeze-drying process was performed at −80 °C and 0.03 mTorr; depending on the section, the procedure was maintained for 24–72 h. After a minimum of 12 h of rest in a desiccator, the dried sections were transformed into powder using a food homogenizer and sieved through a 200-mesh sieve to achieve an approximate particle size of 0.074 mm, except for seeds, which were sieved through a 40-mesh sieve to a particle size of 0.420 mm. For oven drying, fresh fruit samples (pulp, peel, and seeds) were placed in a convection oven at 50 °C. The drying duration varied between 16 and 24 h depending on the specific section and continued until a constant weight was achieved. Each section was replicated thrice.

For extract obtention, the lyophilized, oven-dried, and freshly milled samples were then subjected to solvent-mediated extraction. Briefly, ethanol (EtOH) 70% was added to the sample in proportion to the total dry solids content to be processed to obtain a 1:20 (solid–liquid) ratio for a final volume of 50 mL and homogenized in a vortex. Samples were incubated in a Benchmark SB-12L orbital shaker (Sayreville, NJ, USA) at 45 °C and 150 rpm for 2 h. After shaking, the samples were cooled in an ice bath for 10 min. Once the extraction was complete, the tubes were centrifuged at 2500 rpm, for 15 min at 4 °C utilizing a Gyozen 158OR centrifuge (Gimpo, Republic of Korea). The supernatant was recovered in Falcon tubes. The remaining pellet was washed twice with solvent (EtOH 70%) to 15 mL, and each was then centrifuged under the same conditions previously used. The obtained extracts were filtered using a 0.45 µm membrane filter. The filtered extracts were then placed in an amber ball flask to remove the solvent utilizing a Büchi rotavapor R-100 (New Castle, DE, USA). The water bath was set at 40 °C and the rotation speed was set at 5 rpm for 20 min. The recovered extract was stored in a 50 mL Falcon tube at 80 °C for at least 72 h. After ultra-congelation, the remaining water was removed from the extracts using a freeze dryer at −80 °C and 0.03 mTorr. The lyophilized extracts were stored at −20 °C until use. The entire extraction process was carried out under dark conditions with fruits after 3 post-harvest days, and the samples were protected to protect the extracts from light and oxygen exposure.

### 4.3. Bromatological Analyses

The bromatological analyses of samples from chicozapote fruits were performed following the Association of Official Agricultural Chemists (AOAC) methods. The titratable acidity and pH of samples were investigated via the AOAC 942.15 and 981.12 methods, respectively. The soluble solids and moisture content of samples were studied following the AOAC 932.12 and 920.151 methods, respectively. The TDF, IDF, and SDFP of samples were determined following the AOAC 2011.25 method. The protein, ash, and fat content of samples were analyzed considering the AOAC methods 920.152, 940.26, and 960.39, respectively.

#### 4.3.1. Size, Weight, and Color Evaluation

The diameter and length of samples were measured using a Vernier caliper. The individual weights of samples were determined using an analytical balance. Color analysis was performed using a CR-400 colorimeter (Konica Minolta Sensing, Inc., Tokyo, Japan). Prior to sample evaluation, the colorimeter was calibrated against a standard calibration tile. The pulp, peel, puree, and powder samples were positioned beneath the optic sensor of the colorimeter, and CIE color values in terms of ‘L*’, ‘a*’, and ‘b*’ was recorded. Comparably, chroma (C*) and hue (*h_ab_*) values were assessed from the retrieved data, which were calculated following Equations (1) and (2), respectively. In Equation (1), C* is the chroma value, a* indicates the color coordinates from red (+*a*) to green (−*a*), and b* indicates the color coordinates from yellow (+*b*) to blue (−*b*). All experiments were performed in triplicate.

(1) $$C^{*}={\left(a^{*2}+b^{*2}\right)}^{0.5}$$

(2) $$h_{ab}=arctan\;\frac{b^{*}}{a^{*}}$$

Equations: determination of C* (1) and *h_ab_* (2) for CIE color values.

#### 4.3.2. Titratable Acidity, pH, and Maturity Index

The titratable acidity (%) was calculated using Equation (3), and the results were expressed as g malic acid/100 g of dry solids. Malic acid was utilized as a reference since it is the predominant acid in chicozapotes, with levels ranging from 0.48% to 1.36%.

(3) $$\%\;Total\;tritratable\;acidity={\left(a^{*2}+b^{*2}\right)}^{0.5}$$

### 4.4. Determination of the Content of Bioactive Compounds TPC and TFC

#### 4.4.1. TPC Assay

As published [57], a curve of GA, consisting of 10 data points ranging from 1 to 100 µg/g GA, was constructed to analyze the TPC through the Folin–Ciocalteu (FC) assay. The retrieved calibration curve is presented in the Supplementary Materials section. The FC solution was prepared at a concentration of 0.2 N, while the Na_2_CO_3_ solution was prepared at 0.5 N. Each 60 µL of curve point, blank, or sample was combined with 960 µL of distilled water and 60 µL of Folin–Ciocalteu solution. After incubation for 3 min, 60 µL of Na_2_CO_3_ solution was added. The mixture was incubated in the dark for 90 min. Next, 300 µL of the final mixture was added to each well of a 96-well plate in triplicates. Finally, the absorbance was measured at 765 nm, and the TPC was calculated in milligrams of GAE per gram of DW. The experiment was executed in triplicate.

#### 4.4.2. TFC Assay

The TFC of samples was evaluated as reported [58]. Initially, aluminum trichloride (AlCl_3_), sodium nitrite (NaNO_2_), and sodium hydroxide (NaOH) solutions were prepared at a final concentration of 10% (*w*/*v*), 5% (*w*/*v*), and 1 M, respectively. The experiment was performed by combining 150 µL of samples with 600 µL of distilled water and 45 µL of the NaNO_2_ solution. This mixture was kept under incubation for 5 min. Then, 45 µL of the AlCl_3_ solution was added, and the mixture was incubated for another 6 min. After this, 300 µL of the NaOH solution was incorporated. Finally, 300 µL of the mixture was dispensed into a 96-well plate (per well). The absorbance was then measured at 510 nm, and the TFC was calculated as milligrams of QuE per gram of DW. The calibration curve of Qu was obtained with 10 curve points between 1 and 100 µg/g, and it is illustrated in the Supplementary Materials Section. The experiment was performed in triplicate.

### 4.5. GC-FID Analysis of Methyl Esters

The methyl esters of fatty acids of chicozapote samples were separated using a Hewlett Packard 6890 (Herndon, VA, USA) gas chromatograph integrated with an automatic injector 7683. Considering published reports [59], samples were subjected, weighed, and subjected to a methylation process as reported [60], which consisted initially on freeze-drying samples, placing 0.5 g of samples into test tubes, adding 3 mL of 10% methanolic 1.5 N HCl, vortexing, heating in a water bath at 90 °C for 2 h, and cooling stages, together with the addition of 1 mL of hexane, 10 mL of 6% potassium carbonate (K_2_CO_3_), centrifuging for 5 min, and transfer into 2 mL vials. When methylated, samples were injected into a silica capillary column (SUPELCO SP-2560; 100 m × 0.25 mm × 0.25 µm thickness). Helium was used as the carrier gas at a 1 mL/min flow rate. The injector and FID detector temperatures were 250 °C and 260 °C, respectively. The separation was carried out by injecting 1 µL of the sample with a split ratio of 1:10 into the column at the following gradient: 140 °C for 2.95 min, increasing to 210 °C with a speed of 3 °C/min in a time of 0.0 min, subsequently to 235 °C with a speed of 0.7 °C/min maintaining it for 0 min. Individual components from fatty acids were identified by comparing their retention times with standard fatty acid methyl esters (FAME) Mix C4-C24 1000 μg mL^−1^ (SUPELCO STD catalog No. 18919-1AMP). The total area of the peaks assessed their relative percentage.

### 4.6. Strains, Culture Media, Antibacterial Assay, and Plate Counting

#### 4.6.1. Strains and Culture Media

The antibacterial activity of chicozapote samples was tested against a panel of Gram-negative and Gram-positive bacteria. Gram-negative bacteria included *E. coli* (ATCC 25922), *P. aeruginosa* (ATCC 27853), *S. arizonae* (ATCC 13314), and *K. pneumoniae* (ATCC 700603), whereas Gram-positive strains comprehended *S. aureus* (ATCC 25923) and *E. faecalis* (ATCC 29212). The bacterial strains were cultured in nutrient agar and incubated at 37 °C for 24 h. Then, they were transferred into 5 mL of fresh cation-adjusted Mueller–Hinton broth (CHMB), and incubated at 37 °C for 4–6 h under orbital shaking in an Infors HT Ecotron (Infors AG, Bottmingen, Switzerland) at 225 rpm.

#### 4.6.2. Antibacterial Assay

The antibacterial activity of samples was analyzed via the MIC assay as published [61]. Briefly, chicozapote samples were evaluated at final concentrations of 200, 300, 500, 700, and 1000 µg/mL together with bacterial strains adjusted to 0.5 according to the McFarland scale utilizing g a sterile 96-well microplate at a final volume of 200 μL of CHMB. The MIC was determined by determining the absorbance of wells at 600 nm with an SP-UV1100 spectrophotometer (DLAB Scientific Co., Ltd., Beijing, China), where no growth was observed concerning the absorbance increase. Treatment with ciprofloxacin was appraised as the positive control. All experiments were executed in triplicate.

#### 4.6.3. Plate Counting

The MIC of samples was confirmed through plate counting. Briefly, 10 μL of wells from the MIC assay was removed and dispensed into sterile Eppendorf tubes containing 990 µL of sterile CMHB. After this, the suspension was diluted 1:10 utilizing CMHB, 100 μL was plated onto nutrient agar, and incubated at 37 °C for 24 h. Colonies were counted using a J-3 110 v Luzeren counter, and the effect of treatment was assessed considering Equation (4); here, *N* represents the number of colony-forming units per milliliter, *C* constitutes the number of colonies per plate, *DF* appears for the dilution factor of the 1:10 dilution, and *V* is associated with the inoculated volume on the plate.

(4) $$N=\frac{C\;*DF}{V}$$

### 4.7. Analysis of Antibacterial Activity In Silico

#### 4.7.1. Data Collection

The three-dimensional structures of the most relevant metabolites were obtained from PubChem (https://pubchem.ncbi.nlm.nih.gov/; URL accessed on 22 October 2024). Subsequently, energy minimization was conducted using the Chem3D software with a molecular mechanic force field (MM2), followed by a geometric optimization with the Merck Molecular Force Field (MMFF94), including a dielectric constant of ε = 80.0, which represents a solvated environment. This process yielded the input files required for molecular docking. The crystal structures of DNA gyrase B from each bacterial strain were obtained from the Protein Data Bank (PDB, https://www.rcsb.org; URL accessed on 22 October 2024), with priority given to those with a structural resolution of less than 2.5 Å and, where possible, co-crystallized with an inhibitor. The selected PDB codes were 7DPS (*E. coli* GyrB ATPase domain in complex with Methyl 4-hydroxycinnamate), 6TCK (crystal structure of the ATP binding domain of *S. aureus* GyrB complexed with ULD-2), 8J9T (crystal structure of gyrase of S. enterica), 8BN6 (*P. aeruginosa* DNA gyrase B ATPase subdomain complexed with EBL3021), 4K4O (the DNA gyrase B ATP binding domain of *E. faecalis* in complex with a small molecule inhibitor), and finally, the AF-A0A0H3GXR6-F1 model, obtained by AlphaFold (https://alphafold.ebi.ac.uk/entry/A0A0H3GXR6; URL accessed on 22 October 2024), was chosen as the structure of the DNA gyrase subunit B of *K. pneumoniae*.

#### 4.7.2. Molecular Docking

The preparation of the protein structures before molecular docking entailed the elimination of the ligands and surrounding water molecules in the protein. This was conducted in a manner that maintained only those water molecules that were structurally conserved in the cavity of the recognition site and those that were relevant to the formation of the DNA gyrase B—inhibitor complex. Moreover, all non-functional organic ions and molecules were eliminated from the crystalline complexes. Finally, protein structures were energetically minimized using Chimera 1.17 software (https://www.rbvi.ucsf.edu/chimera/; URL accessed on 22 October 2024) by applying the AMBER ff14SB force field. Affinity energy calculation was performed using Auto Dock Vina software 1.2.0. (https://vina.scripps.edu/; URL accessed on 22 October 2024). Protein and ligand parameterization was performed with MGLTools 1.5.6 (https://ccsb.scripps.edu/mgltools/1-5-6/; URL accessed on 22 October 2024), adding Kollman charges and Gasteiger charges, respectively. A focused docking strategy was employed, whereby the center of the grid box was defined by the orientation coordinates of Asp73 in 7DPS and 8J9T, Asp74 in AF-A0A0H3GXR6-F1 (AFoldKp), Asp75 in 4K4O and 8BN6, and finally, Asp81 in 6TCK. In all cases, the dimensions of the grid box were maintained at 30 × 30 × 30 Å, the charges were fused, nonpolar hydrogens were removed, the position of the structurally conserved water molecules was preserved, and the generation of up to ten distinct binding modes was permitted. The results are expressed in terms of affinity energy in kcal/mol. The graphical representation of the binding mode was processed in ChimeraX (https://www.cgl.ucsf.edu/chimerax/; URL accessed on 22 October 2024), while the two-dimensional diagrams of molecular interactions of the protein–metabolite complex were generated in Discovery Studio 2021 (https://www.3ds.com/products/biovia/discovery-studio; URL accessed on 22 October 2024).

### 4.8. Antioxidant Assay

According to previous protocols of our research group [62], the antioxidant activity of chicozapote samples was analyzed via the DPPH and ABTS assays, respectively. For the DPPH assay, a 1 mM solution was made by dissolving 4 mg of DPPH reagent in technical-grade ethanol, which was stirred moderately for 2 h in a dark environment. Afterward, 200 µL of the resultant solution was added to each well of a 96-well plate and mixed with chicozapote samples at concentrations of 200, 300, 500, 700, and 1000 µg/mL. The plate was kept in the dark for 30 min at room temperature. Absorbance for each well was then measured at 517 nm using a Multiskan Sky microplate reader (Thermo Scientific, Waltham, MA, USA). For the ABTS assay, a stock solution was prepared by dissolving 19.7 mg of ABTS reagent and 186.2 mg of K_2_S_2_O_8_ in 50 mL of distilled water. This mixture was stirred moderately for 1 h in a dark setting. In a 96-well plate, 200 µL of the prepared solution was added to each well together with chicozapote samples at 200, 300, 500, 700, and 1000 µg/mL. The absorbance of wells was analyzed utilizing the same microplate reader. Treatment with Qu at the same concentrations was considered as the positive control. All experiments were performed in triplicate.

### 4.9. In Vivo Toxicity

The *in vivo* toxicity of the obtained chicozapote samples was studied against *A. salina* nauplii as reported [63]. Briefly, dried cysts of *A. salina* were placed in a container containing 35 g of artificial sea salt dissolved in 1 L of distilled water. This container was maintained at 28–30 °C with vigorous aeration and continuous illumination for 48 h. Once the nauplii hatched, 250 µL of them was added to each well of a 96-well plate, along with chicozapote samples at concentrations of 200, 300, 500, 700, and 1000 µg/mL. The survival of the nauplii was monitored over 48 h using an inverted microscope (Leica DMi1, Wetzlar, Germany) equipped with a FLEXACAM C1 camera and analyzed using Leica software version 3.3.0 (Leica Microsystems, MA, Germany). The retrieved results were compared to treatment with K_2_Cr_2_O_7_, which was appraised as the positive control. Experiments were performed in triplicate.

### 4.10. Statistical Analyses

Significant statistical differences were determined via a one-way analysis of variance (ANOVA) followed by Tukey’s mean separation test. The OriginPro 2023 data processing software was utilized to perform analyses.

## 5. Conclusions

In this work, the pulp, peel, and seeds of chicozapote were dried by lyophilization and oven drying, subjected to extraction with EtOH, and evaluated for their bromatological features, phytochemical content, and *in vitro*, *in vivo*, and *in silico* biological performance. Among the obtained extracts, it was demonstrated that lyophilized extracts from the peel and seeds presented the highest ash, protein, and fat content. It was observed that the TPC and TFC were higher in lyophilized extracts from the pulp and peel. Contrarily, the TCT was predominant in oven-dried extracts from the pulp, peel, and seeds of chicozapote fruits. GC-FID analyses revealed the presence of methyl ester derivatives of myristic acid, pentadecanoic acid, and oleic acid among extracts. However, it was recorded that lyophilized extracts from the peel exhibited the highest content of palmitic acid, linoleic acid, and linolenic acid, which is of great importance due to their capacity to exert a wide range of biological properties with clinical implications. Even though the antibacterial activity of extracts was poor against pathogenic Gram-positive and Gram-negative strains, it was reported that the lyophilized extract from the peel can inhibit growth at MIC values < 396 μg/mL. *In silico* studies demonstrated that polar compounds already reported in chicozapote exhibit high affinity towards the DNA gyrase B of the cultured strains. The antioxidant capacity of extracts was poor against DPPH and ABTS radicals, which can be due to the amount of material used for extract preparation, together with TFC and TPC. Despite their differences, lyophilized and oven-dried extracts are not toxic against *A. salina* nauplii, suggesting their biocompatibility. Combined with these retrieved results, this study validates for the first time the influence of drying methods (lyophilization and oven drying) in the bromatological features, phytochemical content, and *in vitro*, *in vivo*, and *in silico* performance of ethanol extracts from the pulp, peel, and seeds of chicozapote fruits. In addition, it suggests the need for additional approaches to improve their activity, and confirms the hypothesis established at the beginning of this work.

## Figures and Tables

**Table 1:** Water and soluble solids, and titratable acidity and maturity index of chicozapote fruits after 9 post-harvest days. Values are presented as the mean ± SD. Letters A–F represent statistical differences between results.

Parameter	Post-Harvest Days						
	3	4	5	6	7	8	9
Water	74.71 ± 0.00 ^A^	76.26 ± 3.53 ^A^	73.31 ± 0.01 ^AB^	72.65 ± 0.00 ^ABC^	72.54 ± 0.01 ^ABC^	68.5 ± 0.00 ^BC^	68.2 ± 0.01 ^C^
Soluble solids	17.55 ± 1.29 ^D^	19.25 ± 0.43 ^BCD^	19.64 ± 0.96 ^CD^	19.98 ± 0.96 ^ABC^	20.67 ± 0.45 ^AB^	20. 78 ± 0.50 ^AB^	21.91 ± 0.32 ^A^
Titratable acidity	0.45 ± 0.09 ^A^	0.49 ± 0.03 ^A^	0.33 ± 0.03 ^B^	0.21 ± 0.03 ^C^	0.1 ± 0.00 ^CD^	0.095 ± 0.01 ^D^	0.093 ± 0.01 ^D^
Maturity index	35.45 ± 2.61 ^F^	42.25 ± 0.95 ^F^	54.46 ± 2.80 ^E^	91.68 ± 1.33 ^D^	196.91 ± 4.31 ^C^	218.54 ± 5.27 ^B^	230.48 ± 3.45 ^A^

**Table 2:** Effect of distinct drying methods in the yield of chicozapote fruits’ pulp, peel, and seeds. Values are presented as the mean ± SD. Letter A represents statistical differences between results.

Source	Drying Method	Yield (% *w*/*w*)
Pulp	Lyophilization	26.12 ± 0.81 ^A^
	Oven-dried	25.87 ± 0.34 ^A^
Peel	Lyophilization	32.33 ± 1.18 ^A^
	Oven-dried	32.68 ± 0.48 ^A^
Seed	Lyophilization	54.06 ± 0.09 ^A^
	Oven-dried	57.53 ± 2.49 ^A^

**Table 3:** Effect of distinct drying methods in the composition of the pulp, peel, and seeds of chicozapote fruits. Values are presented as the mean ± SD and expressed as g/100 g dry sample. Letters (A and B) in the same row indicate significant group differences.

Sample	LPU	OPU	LPE	OPE	LS	OS
Protein	4.61 ± 0.60 ^A^	4.34 ± 0.76 ^A^	5.26 ± 0.59 ^A^	5.04 ± 0.47 ^A^	5.25 ± 0.22 ^A^	5. 10 ± 0.22 ^A^
Ash	7.62 ± 0.36 ^A^	7.91 ±0.18 ^A^	8.37 ± 0.33 ^A^	7.56 ± 0.35 ^A^	11.40 ± 2.70 ^A^	15.10 ± 2.25 ^A^
Fat	0.78 ± 0.06 ^A^	0.93 ± 0.13 ^A^	0.71 ± 0.01 ^B^	6.43 ± 0.17 ^A^	16.09 ± 0.38 ^A^	10.48 ± 0.30 ^B^
IDF	21.80 ± 1.50 ^A^	17.88 ± 0.54 ^B^	29.6 ± 0.08 ^A^	23.53 ± 0.72 ^B^	59.58 ± 3.56 ^A^	55.02 ± 8.90 ^A^
SDFP	19.50 ± 1.40 ^A^	13.74 ± 1.03 ^B^	22.32 ± 0.97 ^A^	11.51 ± 1.74 ^B^	5.31 ± 0.27 ^A^	3.56 ± 0.35 ^B^
TDF	41.37 ± 3.03 ^A^	31.61 ± 1.56 ^B^	51.92 ± 1.04 ^A^	35.04 ± 1.73 ^B^	64.88 ± 3.83 ^A^	58.57 ± 3.25 ^B^
DC	45.62 ± 5.05 ^A^	59.2 ± 3.63 ^A^	33.74 ± 3.97 ^B^	45.92 ± 5.72 ^A^	7.12 ± 0.12 ^B^	12.02 ± 0.59 ^A^

Abbreviations: LPU, lyophilized pulp; OPU, oven-dried pulp; LPE, lyophilized peel; OPE, oven-dried peel; LS, lyophilized seed; OS, oven-dried seed; IDF, insoluble dietary fiber; SDFP, high-molecular weight soluble dietary fiber; TDF; total dietary fiber; DC, digestible carbohydrates.

**Table 4:** Methyl ester derivatives of fatty acids from fresh, lyophilized, and oven-dried pulp, peel, and seeds of chicozapote fruits. Values are expressed as abundance (%).

Sample	Identified Compounds								
	Myristic Acid	Pentadecanoic Acid	Palmitic Acid	Heptadecanoic Acid	Stearic Acid	Oleic Acid	Linoleic Acid	Arachidic Acid	Linolenic Acid
FPU	N.I.	N.I.	38.48	3.70	6.27	12.34	13.12	2.00	3.93
LPU	N.I.	N.I.	28.38	0.97	6.11	13.20	15.61	2.03	5.93
OPU	N.I.	N.I.	34.27	2.25	6.70	24.80	15.54	0.74	2.68
FPE	N.I.	N.I.	22.12	0.73	2.28	9.53	9.98	2.33	4.40
LPE	N.I.	N.I.	38.50	0.99	5.27	18.51	19.11	0.61	9.59
OPE	N.I.	N.I.	37.32	0.86	6.91	28.33	15.00	0.53	6.12
FS	0.19	0.09	22.25	0.18	0.18	53.00	15.30	0.35	1.50
LS	0.21	0.08	22.86	0.19	0.19	56.17	13.93	0.32	0.31
OS	0.21	0.10	22.10	0.21	0.21	56.05	12.81	0.30	0.25

Abbreviations: FPU, fresh pulp; LPU, lyophilized pulp; OPU, oven-dried pulp; FPE, fresh peel; LPE, lyophilized peel; OPE, oven-dried peel; FS, fresh seed; LS, lyophilized seed; OS, oven-dried seed; N.I., not identified.

**Table 5:** Antibacterial activity of lyophilized and oven-dried pulp, peel, and seeds of chicozapote fruits. Results are expressed as the MIC (μg/mL).

Strain	FPU	LPU	OPU	FPE	LPE	OPE	FS	LS	OS	CIP
*E. coli*	800	800	800	600	396	1000	>1000	>1000	>1000	0.25
*S. aureus*	1000	1000	1000	396	396	1000	>1000	>1000	>1000	1
*S. enterica* spp. *arizonae*	800	800	1000	396	396	800	>1000	>1000	>1000	0.06
*P. aeruginosa*	>1000	>1000	>1000	396	198	1000	>1000	>1000	>1000	0.26
*K. pneumoniae*	>1000	>1000	>1000	396	396	1000	>1000	>1000	>1000	0.04
*E. faecalis*	>1000	>1000	>1000	198	198	1000	>1000	>1000	>1000	5

Abbreviations: FPU, fresh pulp; LPU, lyophilized pulp; OPU, oven-dried pulp; FPE, fresh peel; LPE, lyophilized peel; OPE, oven-dried peel; FS, fresh seed; LS, lyophilized seed; OS, oven-dried seed; CIP, ciprofloxacin.

**Table 6:** Affinity energy of polar phytoconstituents of chicozapote fruits on DNA gyrase B of various bacterial species (kcal/mol).

Compound	*E.coli*	*S. aureus*	*S. enterica*	*P. aureginosa*	*K. pneumoniae*	*E. fecalis*	References
	7DPS	6TCK	8J9T	8BN6	AFoldKp	4K4O	
Catechin	−6.9	−7.7	−8.9	−7.7	−8.2	−8.2	[36]
Chlorogenic acid	−6.9	−7.5	−8.9	−8.0	−7.9	−8.2	[36]
Dihydromyricetin	−7.0	−8.5	−8.8	−7.8	−8.3	−8.6	[36]
Lutein	−4.7	−6.9	−8.2	−7.0	−7.0	−6.5	[2]
Methyl chlorogenate	−6.9	−7.1	−9.0	−7.3	−8.1	−7.7	[36]
Myricitrin	−6.3	−7.8	−10.1	−7.9	−6.7	−8.3	[36,37]
Quercetin	−6.6	−8.4	−8.4	−8.6	−8.2	−8.5	[37]
Syringic acid	−4.0	−5.7	−6.3	−5.6	−6.0	−5.8	[37]
Vanillic acid	−5.0	−5.8	−6.2	−5.8	−5.9	−6.1	[37]

**Table 7:** IC_50_ of lyophilized and oven-dried extracts from chicozapote. Concentrations are expressed in μg/mL.

Sample	DPPH	ABTS
FPU	29,691.90	22,755.81
LPU	27,264.34	50,147.30
OPU	8540.14	38,519.53
FPE	8178.44	36,490.64
LPE	7447.83	103,965
OPE	9343.33	21,132.75
FS	25,550.95	42,073.66
LS	7513.75	101,002.6
OS	3330.20	26,361.94
Qu	<2	<2

## Data Availability

Data generated from this work can be consulted with corresponding authors upon reasonable request.

## Supplementary Materials

The following supporting information can be downloaded at: https://www.mdpi.com/article/10.3390/plants14020216/s1, Figure S1: Calibration curve of gallic acid for TPC assay; Figure S2: Calibration curve of quercetin for TFC assay; Figure S3: GC-FID analysis of FPU; Figure S4: GC-FID analysis of LPU; Figure S5: GC-FID analysis of OPU; Figure S6: GC-FID analysis of FPE; Figure S7: GC-FID analysis of LPE; Figure S8: GC-FID analysis of OPE; Figure S9: GC-FID analysis of FS; Figure S10: GC-FID analysis of LS; Figure S11: GC-FID analysis of OS; Figure S12: Conformational alignment of inhibitors predicted by molecular docking (lime green) and the co-crystallized bioactive conformation (cyan) with DNA gyrase B; Figure S13: Two-dimensional diagram of molecular interactions and energy affinity of metabolites on DNA gyrase from different bacterial species.
