# Supplementary material for: Lyophilized and Oven-Dried Manilkara zapota Extracts: Characterization and In Vitro, In Vivo, and In Silico Analyses
Source: Plants (Basel). 2025 Jan 14;14(2):216. doi: 10.3390/plants14020216 (PMC11768716; doi:10.3390/plants14020216)
Supplement: Supplementary file 1 [file plants-14-00216-s001.zip › plants-3403819-supplementary.pdf]

Article

Lyophilized and Oven-Dried *Manilkara zapota* Extracts: Characterization and *In Vitro*, *In Vivo*, and *In Silico* Analyses

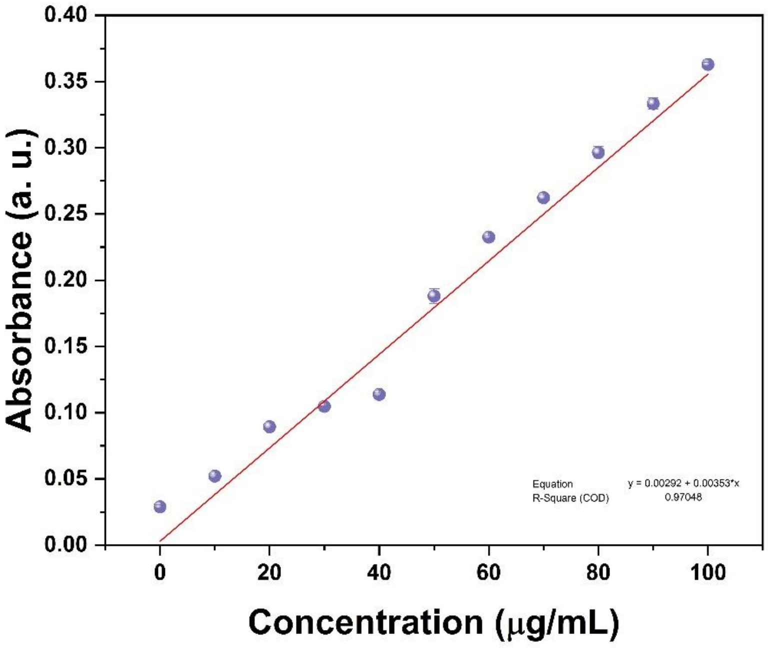

Fig. S1. Calibration curve of gallic acid for TPC assay.

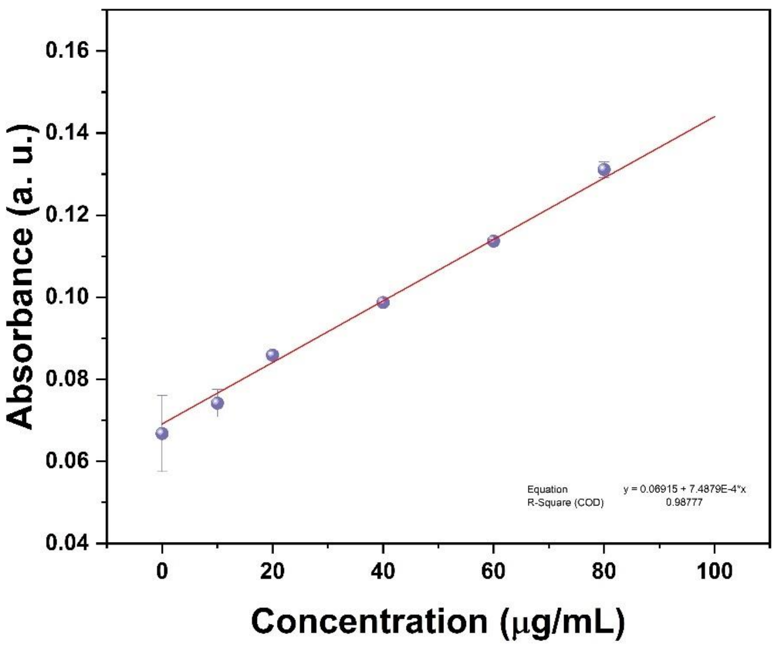

Fig. S2. Calibration curve of quercetin for TFC assay.

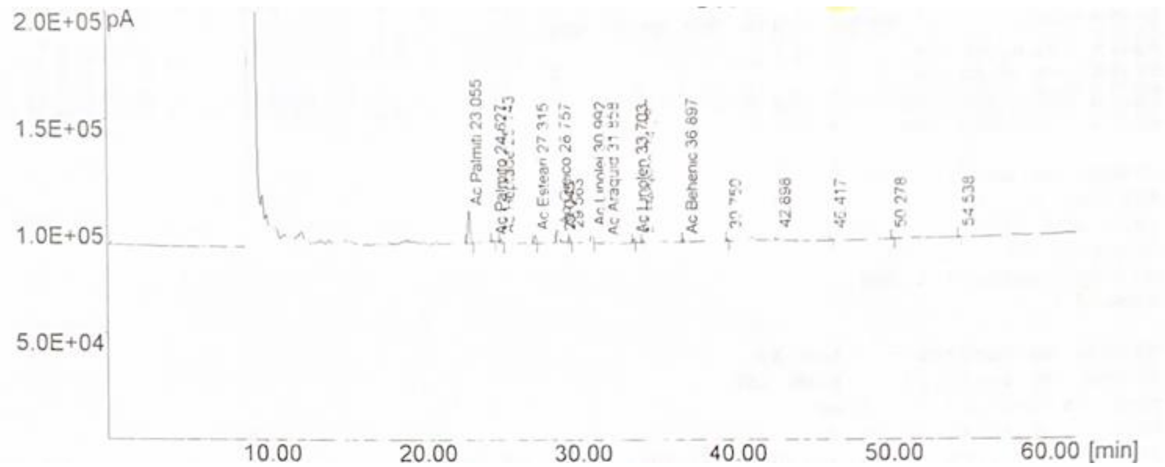

Fig. S3. GC-FID analysis of FPU

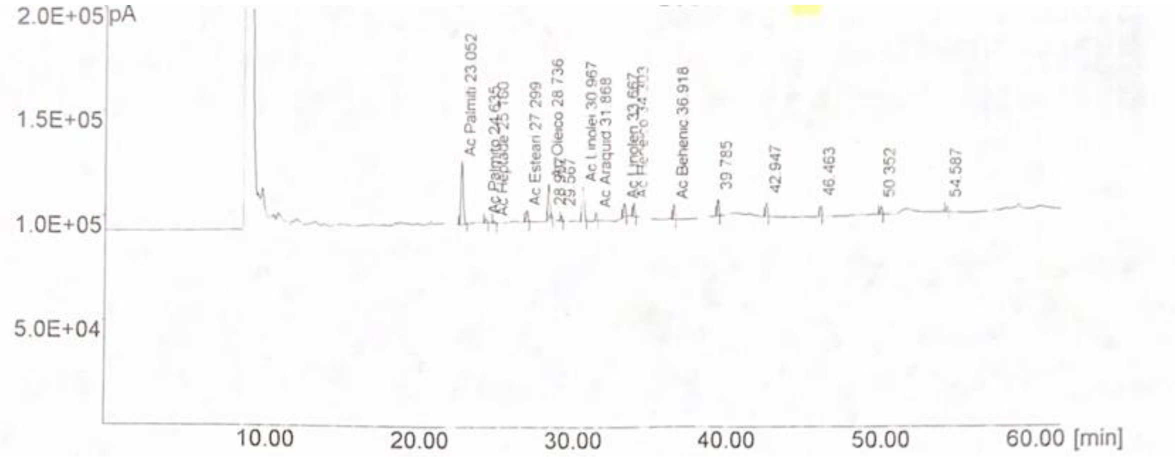

Fig. S4. GC-FID analysis of LPU

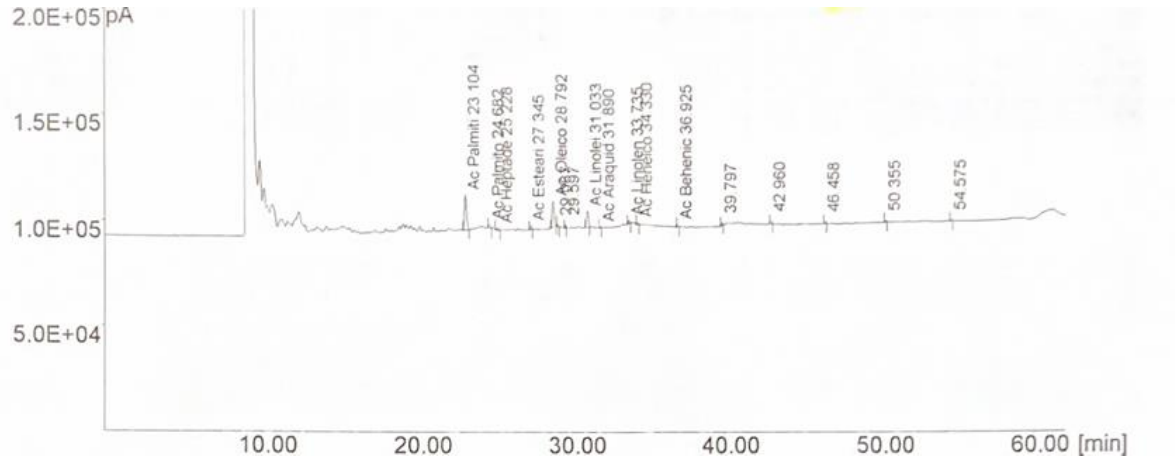

Fig. S5. GC-FID analysis of OPU

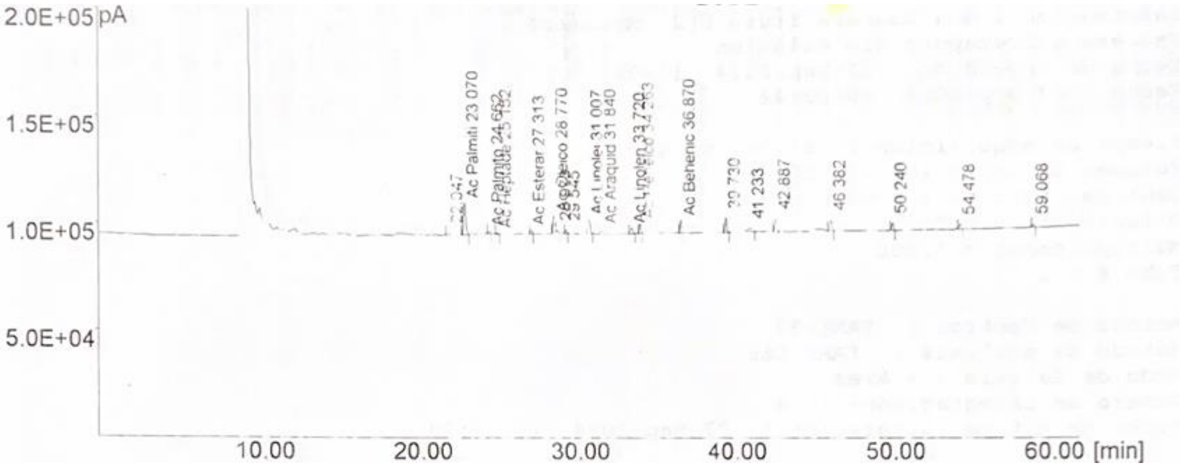

Fig. S6. GC-FID analysis of FPE

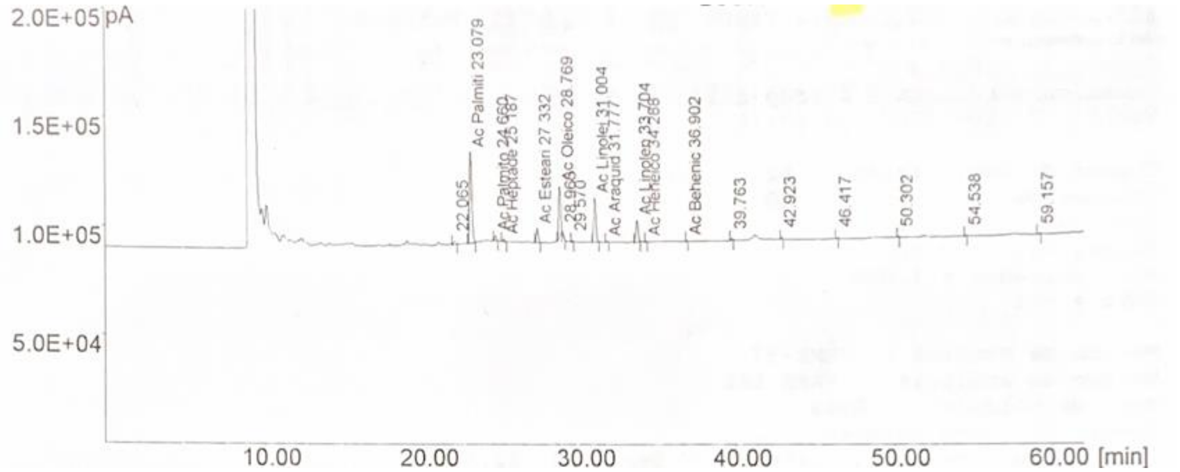

Fig. S7. GC-FID analysis of LPE

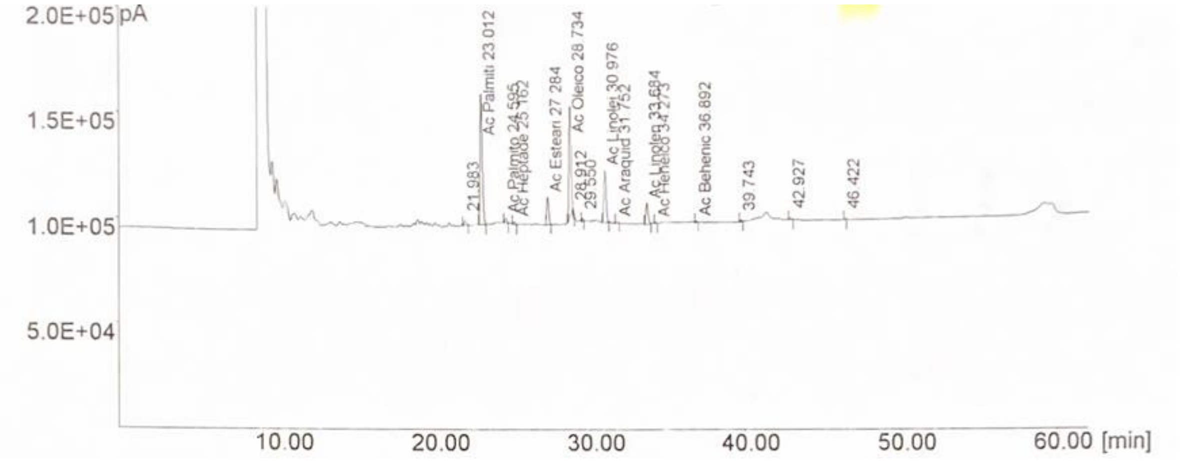

Fig. S8 GC-FID analysis of OPE

.

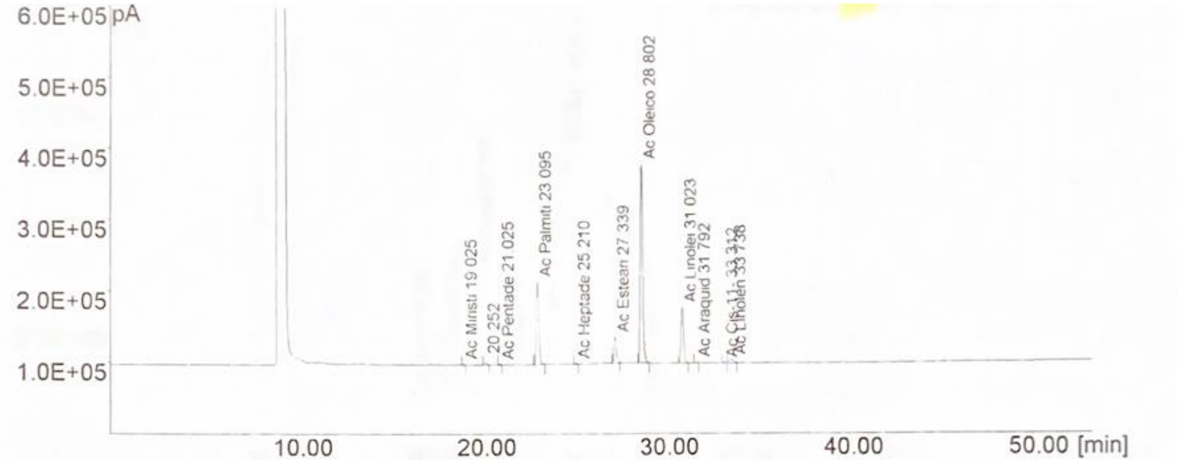

Fig. S9. GC-FID analysis of FS.

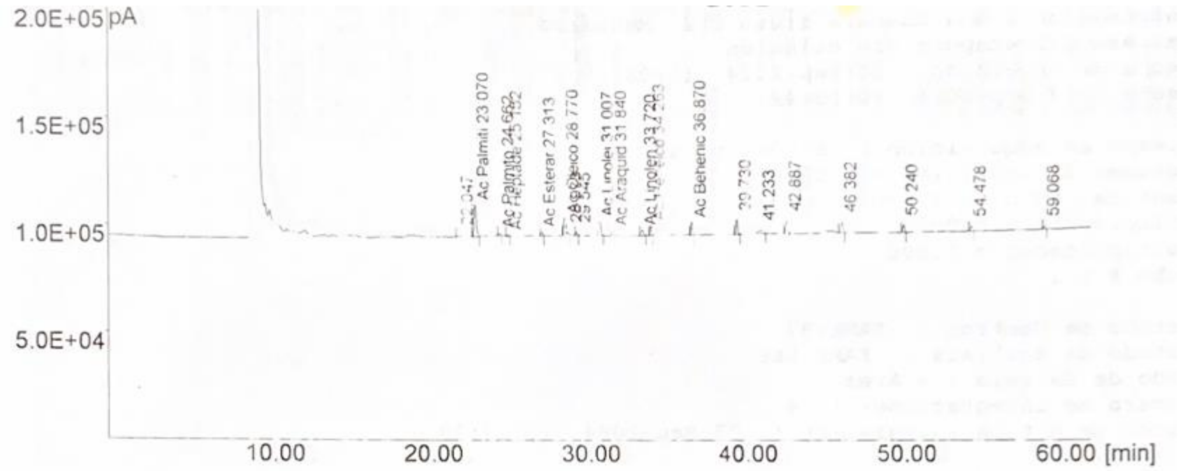

Fig. S10. GC-FID analysis of LS.

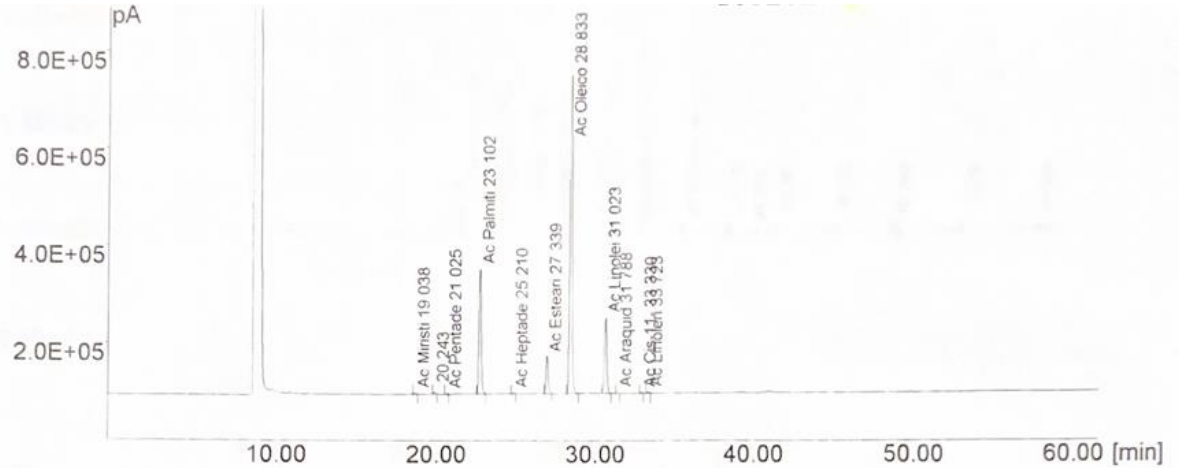

Fig. S11. GC-FID analysis of OS

A. PDB ID: 7DPS; Methyl 4-hydroxycinnamate. ΔG = -5.8 Kcal/mol; RMSD = 0.4676 Å.

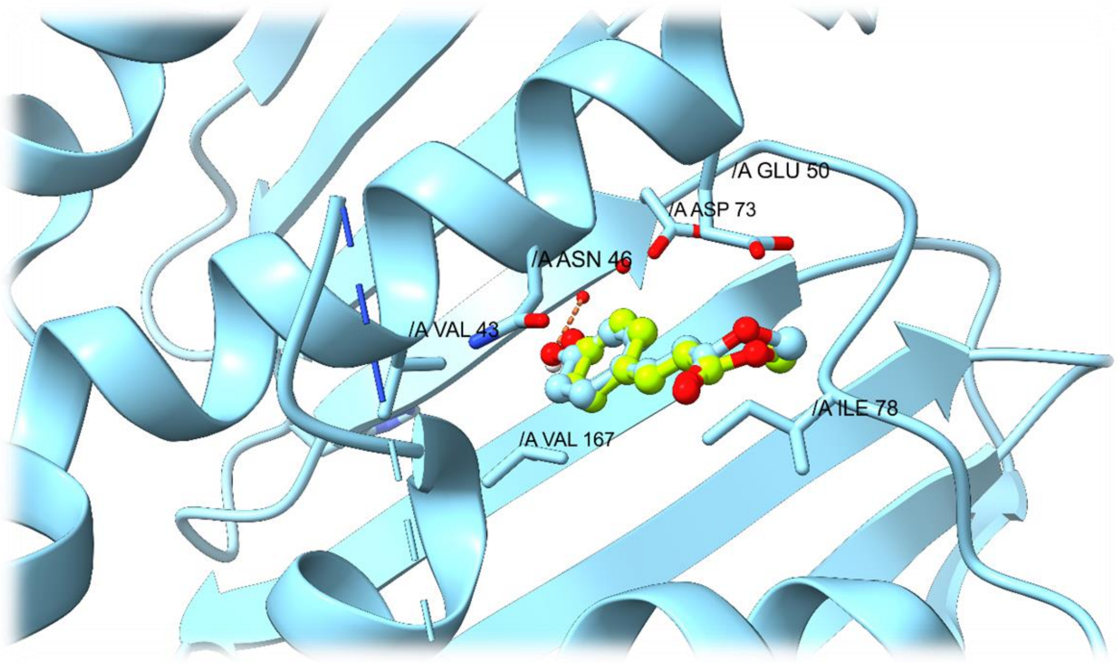

B. PDB ID: 6TCK. 2-[[3,4-bis(chloranyl)-5-methyl-1~{H}-pyrrol-2-yl]carbonylamino]-4-phenylmethoxy-1,3-benzothiazole-6-carboxylic acid. ΔG = -8.7 Kcal/mol; RMSD = 1.5445 Å.

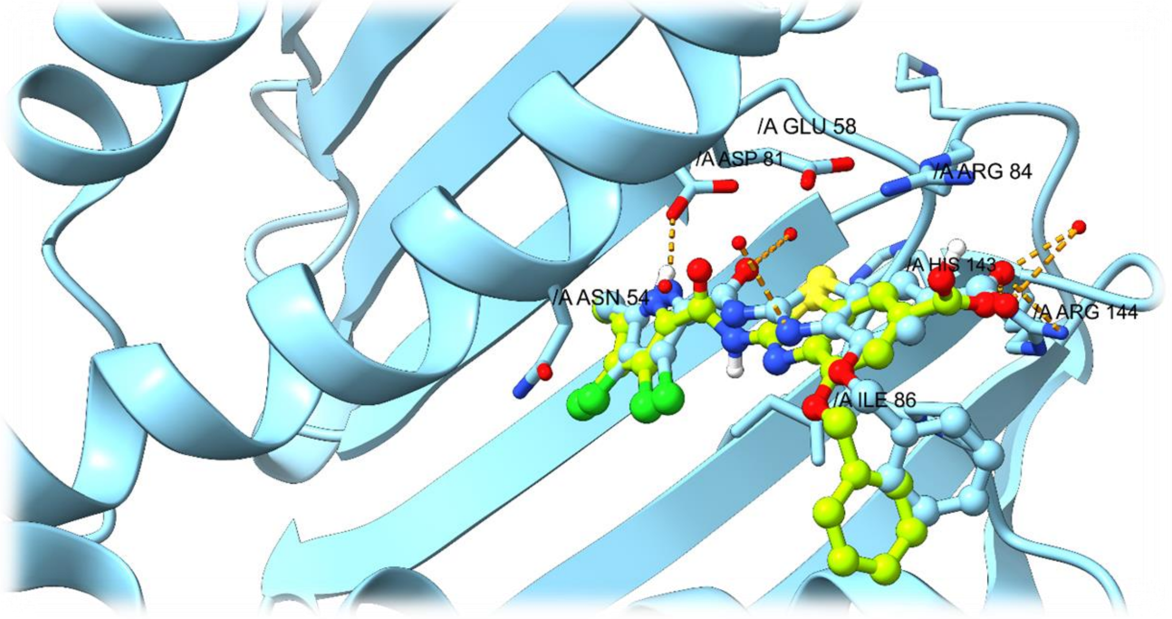

C. PDB ID: 8BN6. 2-[[3,4-bis(chloranyl)-5-methyl-1~{H}-pyrrol-2-yl]carbonylamino]-4-morpholin-4-yl-1,3-benzothiazole-6-carboxylic acid. ΔG = -7.8 Kcal/mol; RMSD = 1.2476 Å.

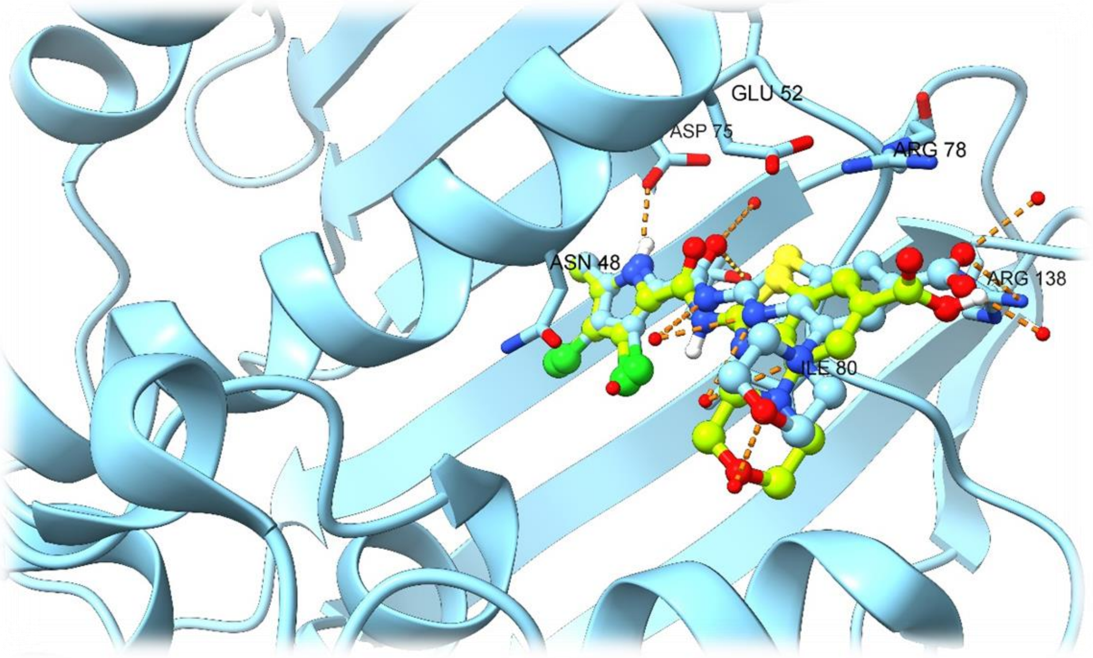

D. PDB ID: 4K4O. 6-F-4-[(3aR,6aR)-hexahydropyrrolo[3,4-b]pyrrol-5(1H)-yl]-*N*-methyl-2-[(2-methylpyrimidin-5-yl)oxy]-9H-pyrimido[4,5-b]indol-8-amine. ΔG = -10.7 Kcal/mol; RMSD = 0.8685 Å.

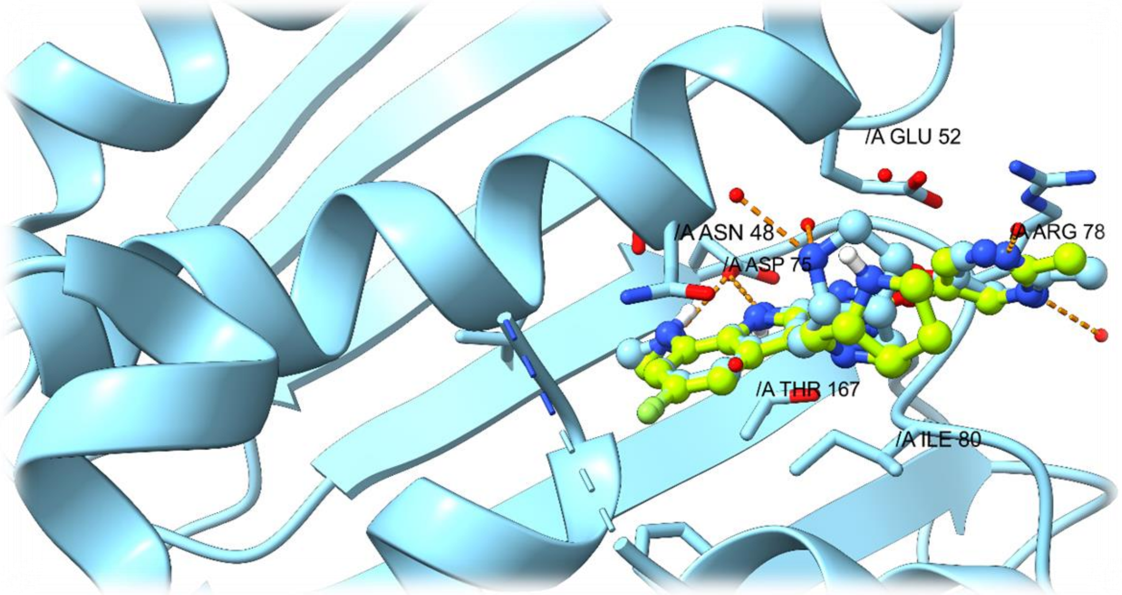

Fig. S12. Conformational alignment of inhibitors predicted by molecular docking (lime green) and the co-crystallized bioactive conformation (cyan) with DNA gyrase B. **A.** Methyl 4-hydroxycinnamate inhibitor in the 7DPS complex; **B.** ULD-2 inhibitor (PubChem CID: 151595514) in the 6TCK complex; **C.** R53 inhibitor (PubChem CID: 167530343) in the 8BN6 complex; **D.** DOO inhibitor (PubChem CID: 66560858) in the 4K4O complex.

2D diagram of molecular interactions of metabolites on DNA gyrase from different bacterial species.

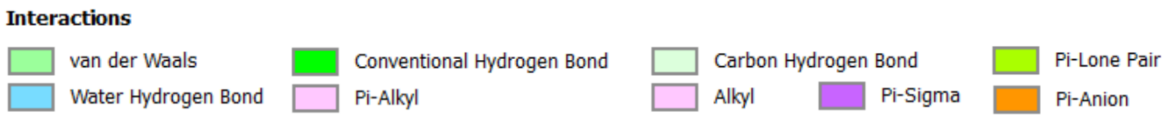

7DPS, *Escherichia coli.*

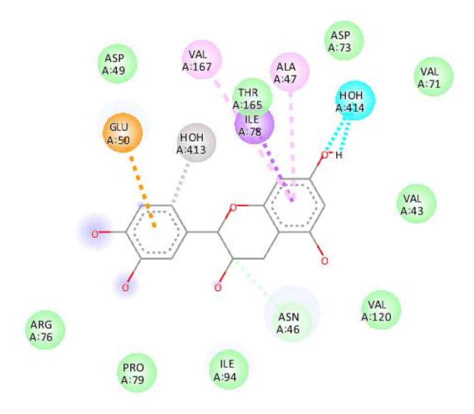

Catechin, -6.9 Kcal/mol

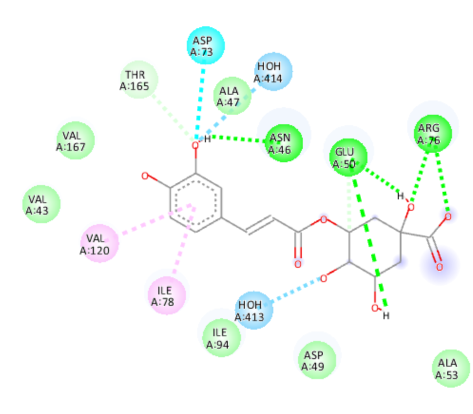

Chlorogenic acid, -6.9 Kcal/mol

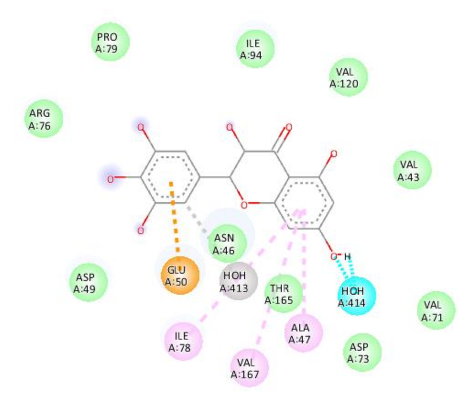

Dihydromyricetin, -7.0 Kcal/mol.

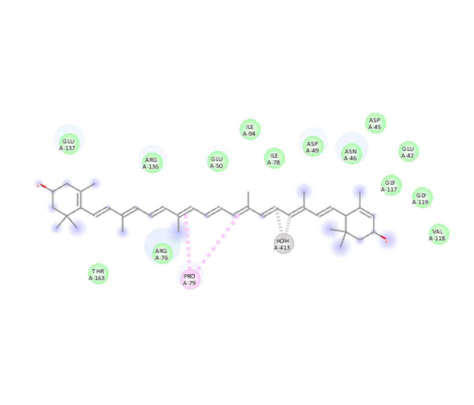

Lutein, -4.7 Kcal/mol

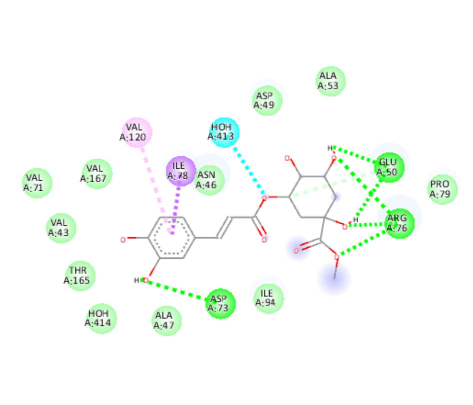

Me-chlorogenate, -6.9 Kcal/mol

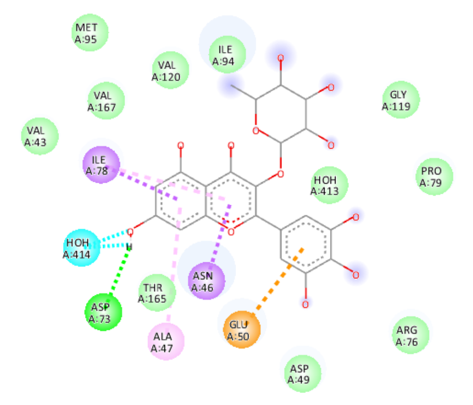

Myricitrin, -6.3 Kcal/mol

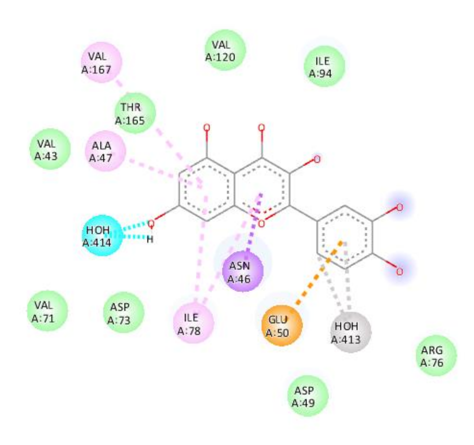

Quercetin, -6.6 Kcal/mol

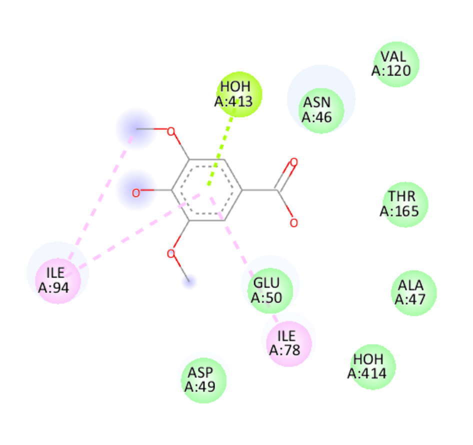

Syringic acid, -4.0 Kcal/mol

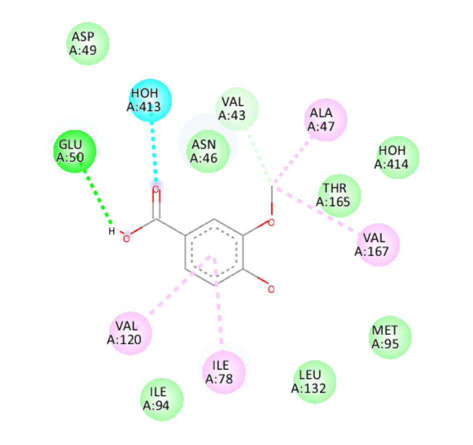

Vanillic acid, -5.0 Kcal/mol

6TCK, *Staphylococcus aureus.*

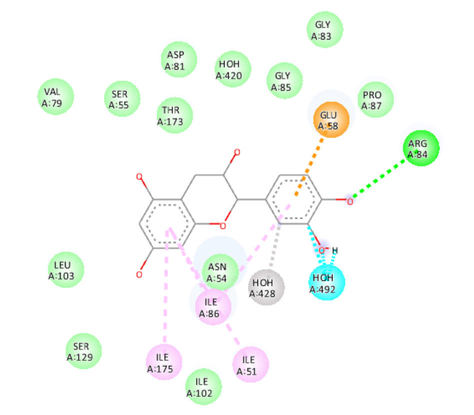

Catechin, -7.7 Kcal/mol

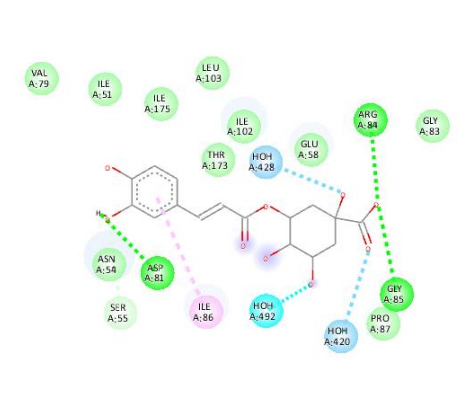

Chlorogenic acid, -7.5 Kcal/mol

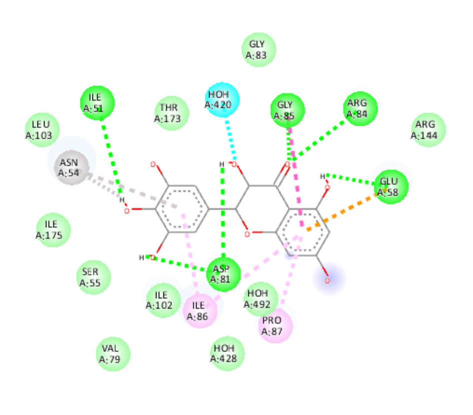

Dihydromyricetin, -8.5 Kcal/mol.

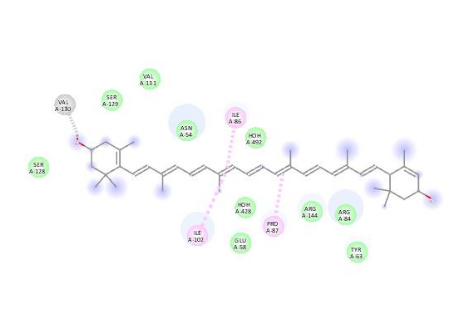

Lutein, -6.9 Kcal/mol

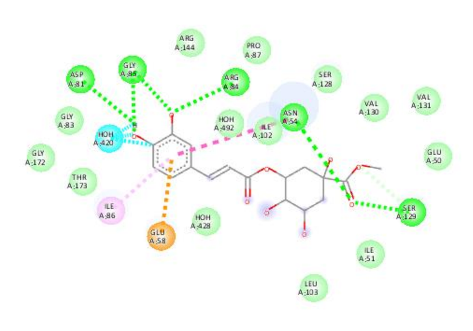

Me-chlorogenate, -7.1 Kcal/mol

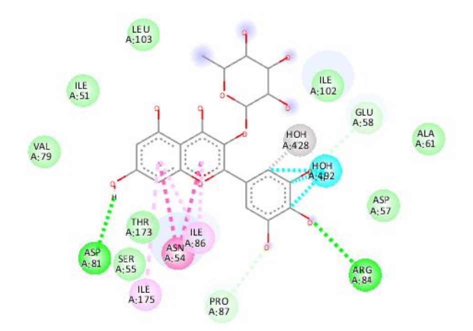

Myricitrin, -7.8 Kcal/mol

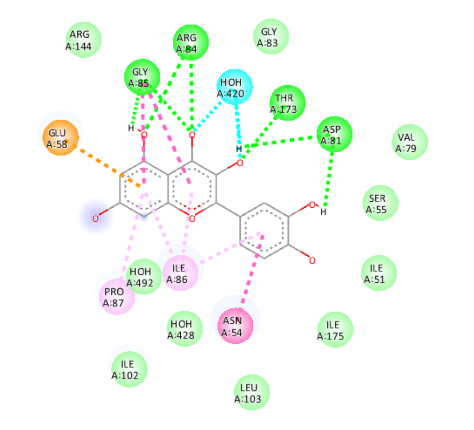

Quercetin, -8.4 Kcal/mol

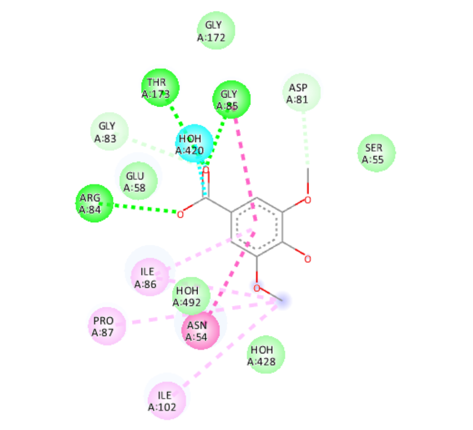

Syringic acid, -5.7 Kcal/mol

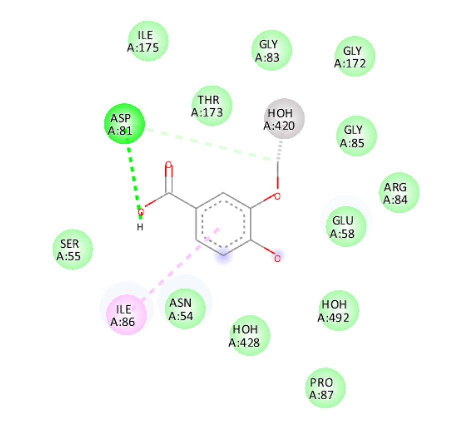

Vanillic acid, -5.8 Kcal/mol

8J9T, *Salmonella entérica.*

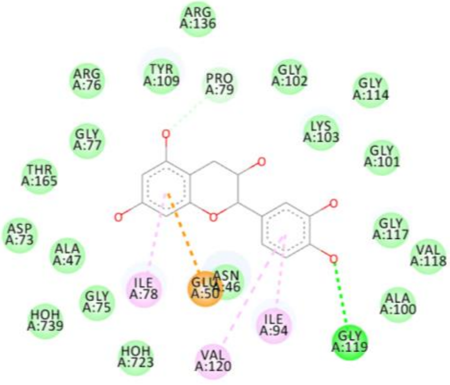

Catechin, -8.9 Kcal/mol.

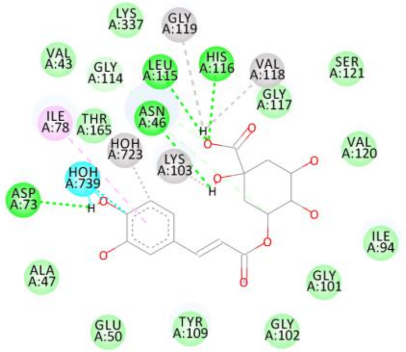

Chlorogenic acid, -8.9 Kcal/mol.

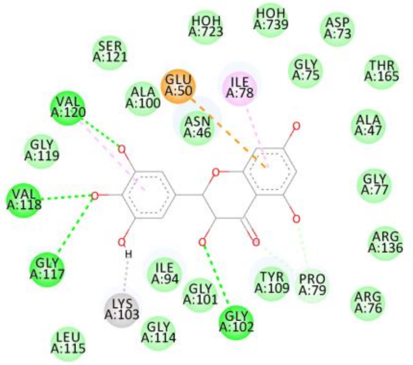

Dihydromyricetin, -8.8 Kcal/mol.

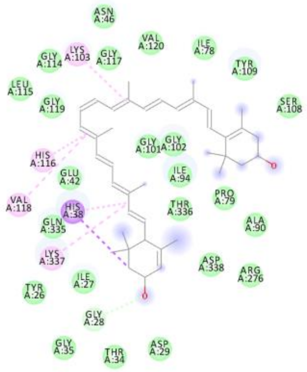

Lutein, -8.2 kcal/mol.

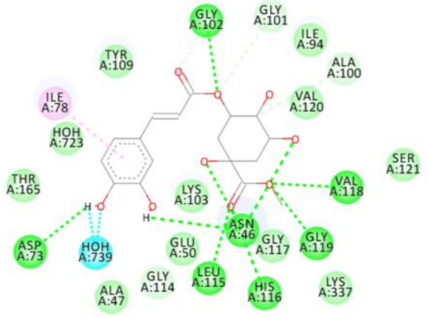

Me-chlorogenate, -9.0 kcal/mol.

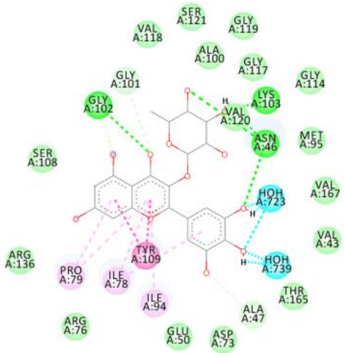

Myricitrin, -10.1 kcal/mol.

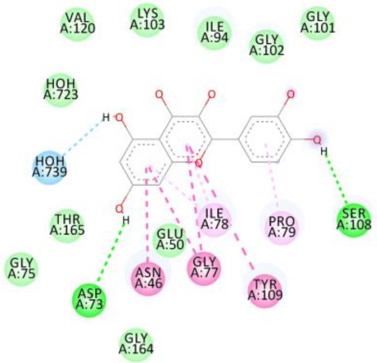

Quercetin, -8.4 kcal/mol.

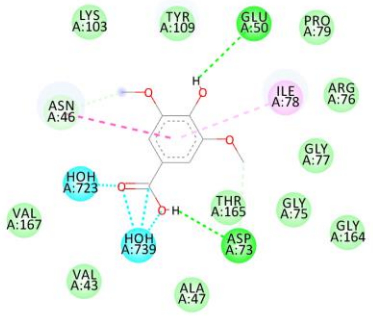

Syringic acid, -6.3 kcal/mol.

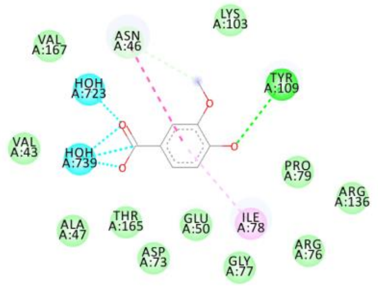

Vanillic acid, -6.2 kcal/mol.

8BN6, *Pseudomonas aeruginosa*.

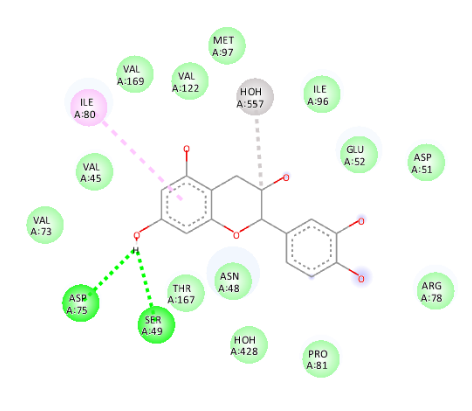

Catechin, -7.7 Kcal/mol

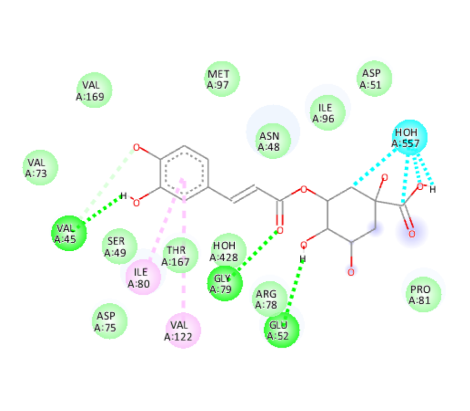

Chlorogenic acid, -8.0 Kcal/mol

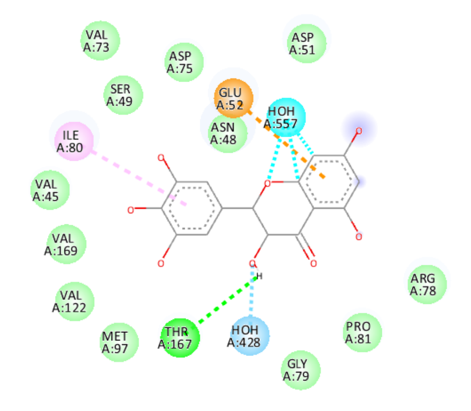

Dihydromyricetin, -7.8 Kcal/mol.

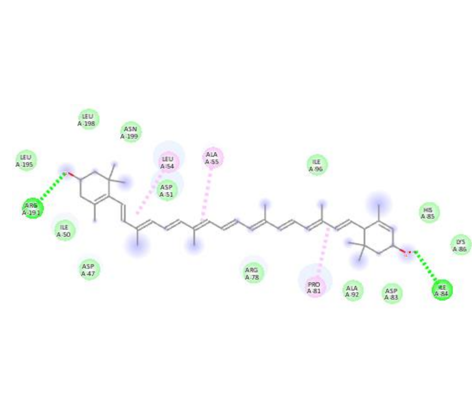

Lutein, -7.0 Kcal/mol

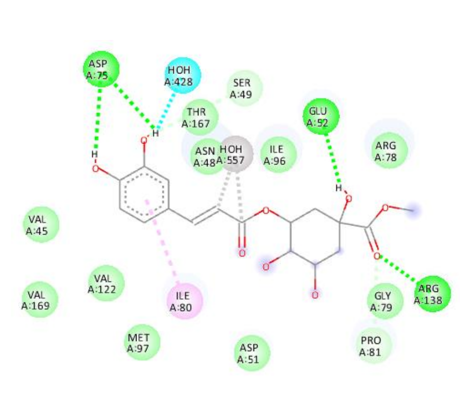

Me-chlorogenate, -7.3 Kcal/mol

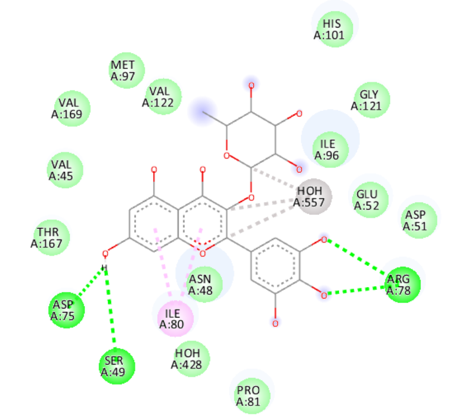

Myricitrin, -7.9 Kcal/mol

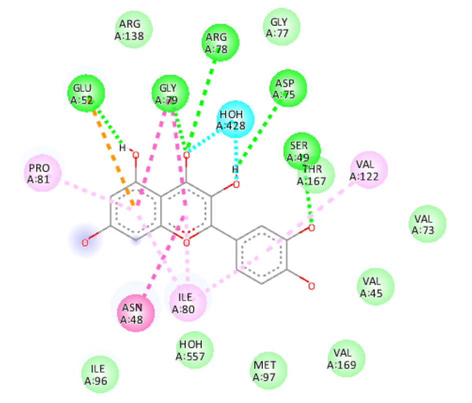

Quercetin, -8.6 Kcal/mol

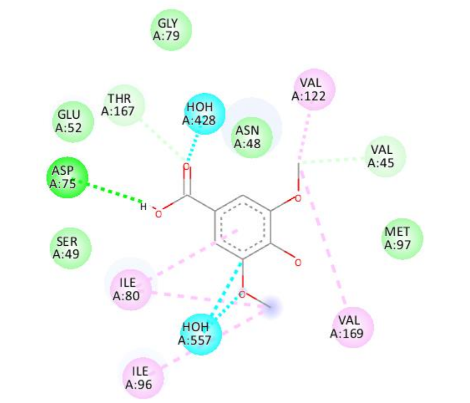

Syringic acid, -5.6 Kcal/mol

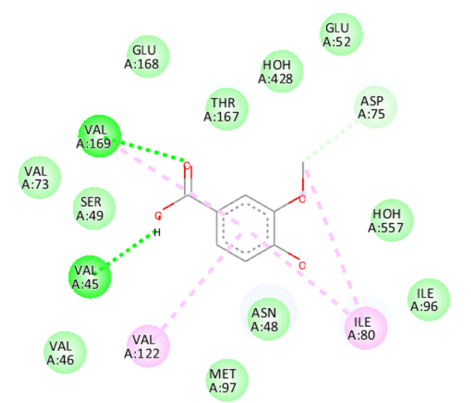

Vanillic acid, -5.8 Kcal/mol

AFoldKp, *Klebsiella pneumoniae*.

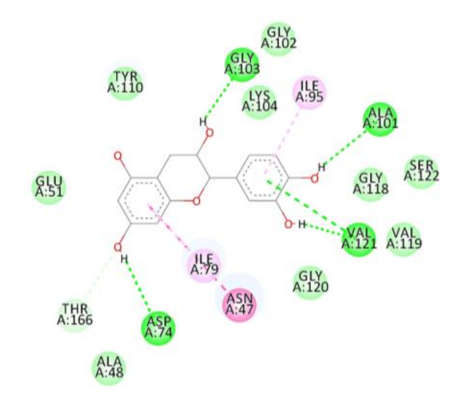	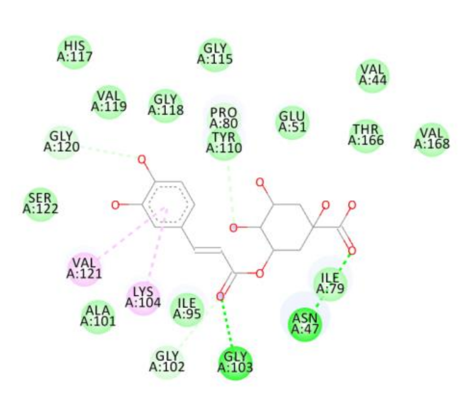	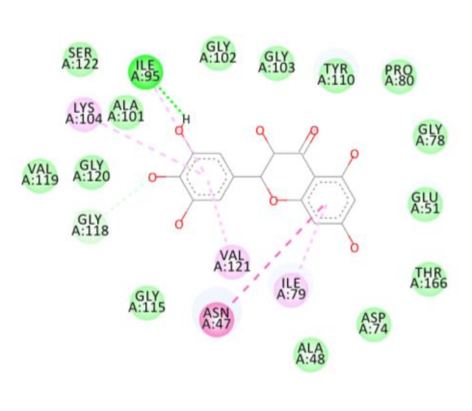
Catechin, -8.2 Kcal/mol.	Chlorogenic acid, -7.9 Kcal/mol.	Dihydromyricetin, -8.3 Kcal/mol.
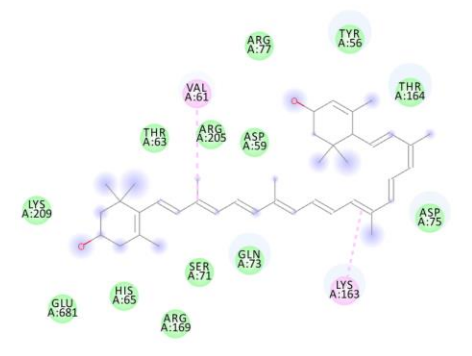	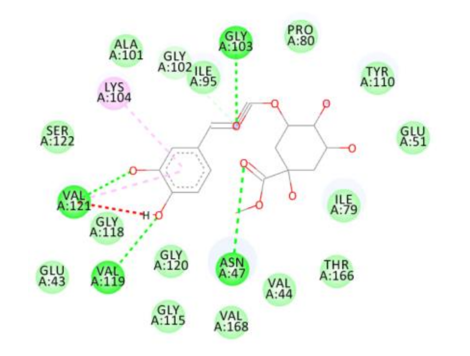	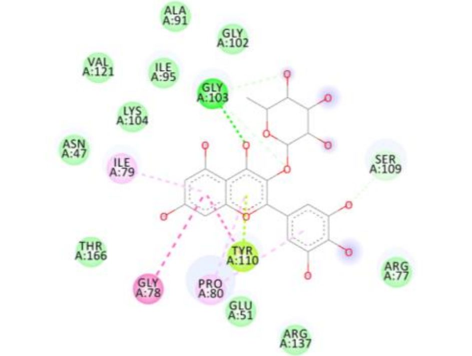
Lutein, -7.0 kcal/mol.	Me-chlorogenate, -8.1 kcal/mol.	Myricitrin, -6.7 kcal/mol.
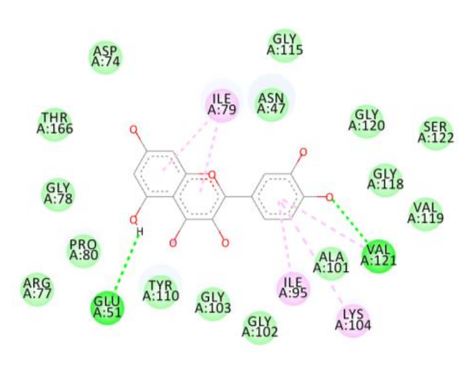	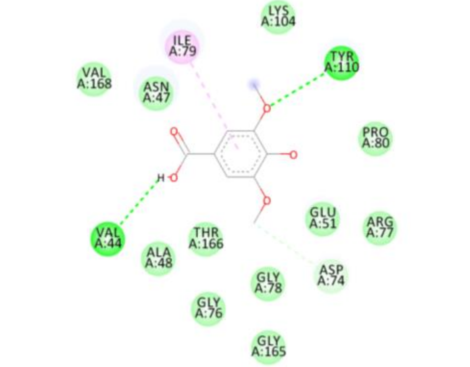	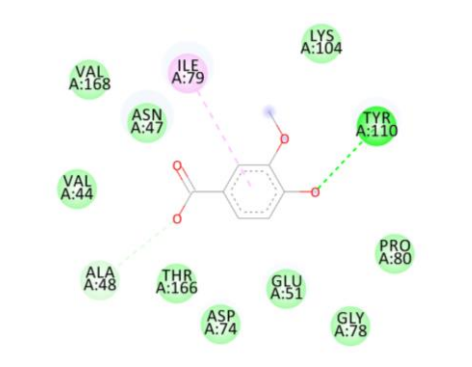
Quercetin, -8.2 kcal/mol.	Syringic acid, -6.0 kcal/mol.	Vanillic acid, -5.9 kcal/mol.

4K4O, *Enterococcus faecalis*.

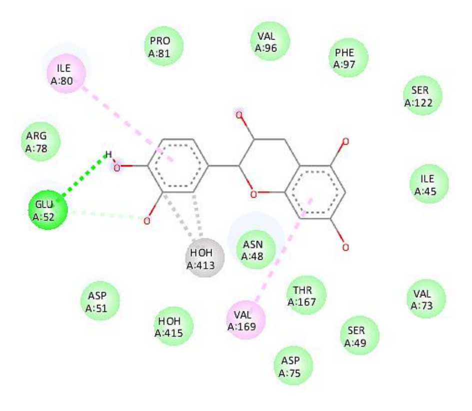

Catechin, -8.2 Kcal/mol

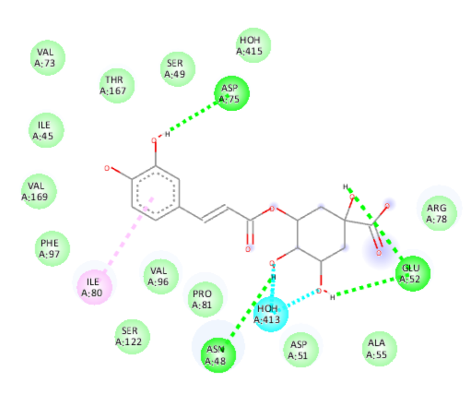

Chlorogenic acid, -8.2 Kcal/mol

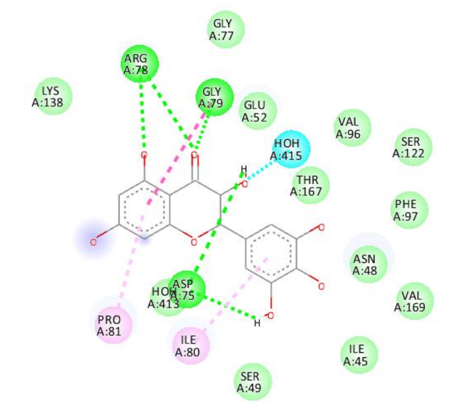

Dihydromyricetin, -8.6 Kcal/mol.

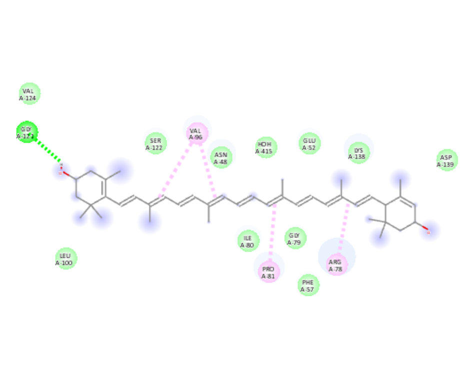

Lutein, -6.5 Kcal/mol

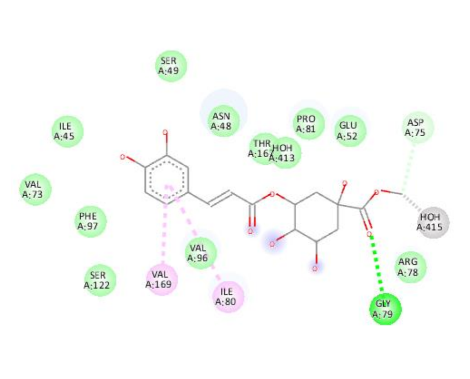

Me-chlorogenate, -7.7 Kcal/mol

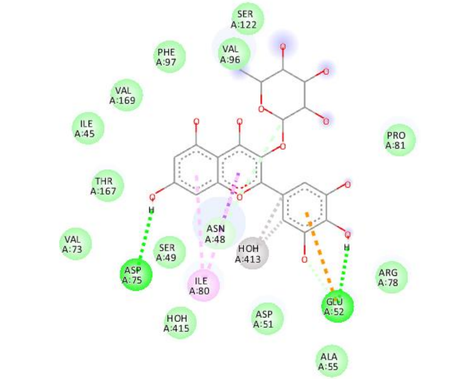

Myricitrin, -8.3 Kcal/mol

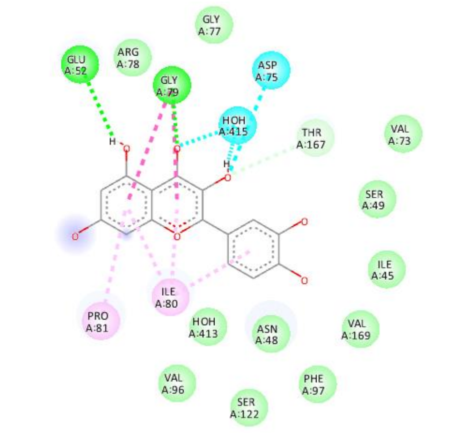

Quercetin, -8.5 Kcal/mol

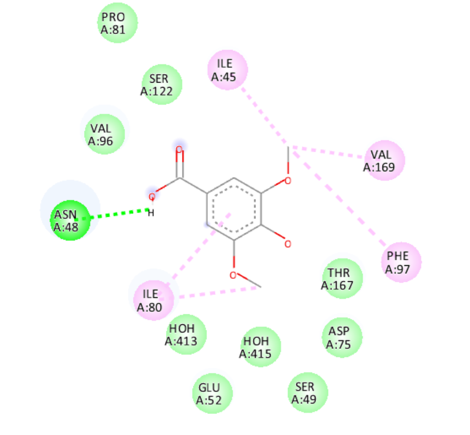

Syringic acid, -5.8 Kcal/mol

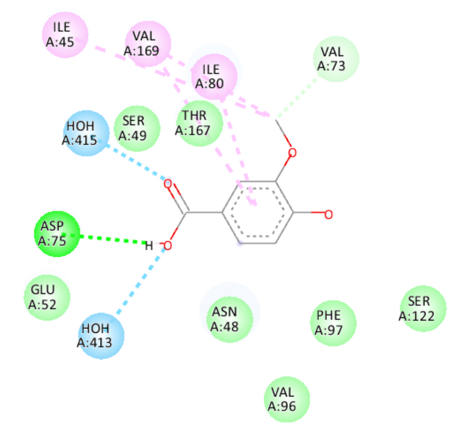

Vanillic acid, -6.1 Kcal/mol

Fig. S13. 2D diagram of molecular interactions and energy affinity of metabolites on DNA gyrase from different bacterial species.
